# Sirtuin inhibition is synthetic lethal with *BRCA1* or *BRCA2* deficiency

**DOI:** 10.1038/s42003-021-02770-2

**Published:** 2021-11-08

**Authors:** Ilirjana Bajrami, Callum Walker, Dragomir B. Krastev, Daniel Weekes, Feifei Song, Andrew J. Wicks, John Alexander, Syed Haider, Rachel Brough, Stephen J. Pettitt, Andrew N. J. Tutt, Christopher J. Lord

**Affiliations:** 1 The CRUK Gene Function Laboratory, The Institute of Cancer Research, London, SW3 6JB UK; 2 Breast Cancer Now Toby Robins Research Centre, The Institute of Cancer Research, London, SW3 6JB UK; 3 Present Address: The Francis Crick Institute, London, NW1 1AT UK

**Keywords:** Homologous recombination, DNA repair enzymes

## Abstract

PARP enzymes utilise NAD^+^ as a co-substrate for their enzymatic activity. Inhibition of PARP1 is synthetic lethal with defects in either *BRCA1* or *BRCA2*. In order to assess whether other genes implicated in NAD^+^ metabolism were synthetic lethal with *BRCA1* or *BRCA2* gene defects, we carried out a genetic screen, which identified a synthetic lethality between *BRCA1* and genetic inhibition of either of two sirtuin (SIRT) enzymes, SIRT1 or SIRT6. This synthetic lethal interaction was replicated using small-molecule SIRT inhibitors and was associated with replication stress and increased cellular PARylation, in contrast to the decreased PARylation associated with *BRCA*-gene/PARP inhibitor synthetic lethality. SIRT/*BRCA1* synthetic lethality was reversed by genetic ablation of either *PARP1* or the histone PARylation factor-coding gene *HPF1*, implicating PARP1/HPF1-mediated serine ADP-ribosylation as part of the mechanistic basis of this synthetic lethal effect. These observations suggest that PARP1/HPF1-mediated serine ADP-ribosylation, when driven by SIRT inhibition, can inadvertently inhibit the growth of *BRCA*-gene mutant cells.

## Abstract (web summary)

Bajrami, Walker et al. investigated the synthetic lethality between BRCA gene defects and inhibition of two sirtuin genes, SIRT1 or SIRT6, which was found to be associated with replication stress and increased PARylation. The authors demonstrated that the SIRT/BRCA1 synthetic lethality was reversed by genetic ablation of PARP1 or HPF1.

## Introduction

PARylation, the addition of poly-ADP-ribose (PAR) chains onto proteins, regulates a diverse set of cellular processes, including the maintenance of genomic stability^1,2^. Poly-ADP-ribosylation is catalysed by a series of structurally related proteins, collectively termed the Poly-ADP-Ribose-Polymerase (PARP) superfamily. PARP enzymes synthesise PAR by adding ADP-ribose moieties onto proteins, using NAD^+^ as a co-substrate^2,3^. Of the 17 PARP superfamily members, PARP1 is the most well studied and is known to play a critical role in DNA repair. PARP1 binds damaged DNA and this interaction stimulates the auto-PARylation of PARP1 and trans-PARylation of many important chromatin factors, such as histones^4,5^.

PARPs are known to modify target proteins at specific residues. Canonically, this occurs at the acidic amino acids (e.g., Asp/Glu)^6^. More recently, however, it has been shown that upon DNA damage, the main form of ADP-ribosylation is *via* serine resides^7–10^. In response to DNA damage, Histone PARyation Factor 1 (HPF1; also known as C4orf27) promotes PARP1-mediated serine ADP-ribosylation of histones by forming a composite active site with PARP1^10^. HPF1 also promotes PARP1 auto-PARylation^7,8^, both of which promote timely DNA repair.

The removal or degradation of PAR chains is also essential for DNA repair^11,12^. The removal of PAR chains is mediated by PAR Glycohydrolase (PARG), which cleaves ribose–ribose bonds in PAR chains^13,14^. An additional PAR hydrolase, ADP-Ribosylhydrolase 3 (ARH3, *ADPRHL2*) also degrades PAR chains, but has greater specificity towards serine ADP-ribosylated (Ser-ADPr) histones^15,16^. Together, these enzymes orchestrate a highly regulated molecular cascade that enables efficient DNA repair^1^.

As for PARP superfamily enzymes, the Sirtuin family of deacetylases utilise NAD^+^ as a co-substrate, but rather than catalysing PARylation, catalyse the deacetylation of target proteins^17^. Of the seven sirtuin family members^18^, SIRT1 and SIRT6 have been shown to regulate aspects of chromosome stability: SIRT1 and SIRT6 defects lead to severe genomic instability, sensitivity to DNA damage-inducing agents, and premature aging in animal models^18–20^. In particular, SIRT1 and SIRT6 have been shown to regulate PARP1 directly, by catalysing the deacetylation of PARP1^19,21,22^. Another major SIRT1 and SIRT6 deacetylation substrate is Histone H3; H3 deacetylation plays a critical role in the remodelling of chromatin architecture following DNA damage^23,24^. Intriguingly, histone acetylation and histone PARylation appear to be mutually exclusive events^25^, possibly suggesting reciprocal biological functions.

Homologous recombination (HR) represents an error-free DNA repair pathway where sister chromatids are used as a template for polymerase-based repair of DNA lesions, such as stalled replication forks^26^. HR is initiated by DNA-end resection, a process that is regulated by the tumour suppressor protein, BRCA1. Single-stranded DNA at resected sites is then bound by the RAD51 DNA recombinase, an event that allows ssDNA strand invasion of undamaged double-stranded DNA on a sister chromatid as a prelude to the use of the sister chromatid DNA as a template. As well as requiring DNA resection, RAD51 loading onto DNA is a BRCA2-dependent process^27,28^. Defects in HR, caused by *BRCA1* or *BRCA2* loss-of-function mutations, lead to genomic instability and predispose to breast and ovarian cancer^29,30^ and also cause synthetic lethality with PARP inhibitors, which, in part at least mediates cytotoxicity by trapping PARP1 on DNA^31,32^. This synthetic lethality is now approved for the treatment of breast, ovarian, prostate and pancreatic cancers^33–36^.

Previously, we demonstrated that depleting cellular NAD^+^ levels using nicotinamide phosphoribosyltransferase (NAMPT) inhibitors enhanced the synthetic lethal effects of clinical PARP inhibitors^37^. Here, we assessed whether other genes implicated in NAD^+^ metabolism were also synthetic lethal in *BRCA*-gene defective cells. To assess this, we performed an RNA interference (RNAi) screen using a panel of genes involved in NAD^+^ metabolism. This identified a PARP1-dependent synthetic lethal interaction between SIRT1/6 and *BRCA1-* or *BRCA2* defects that was characterised by increased PARP1 and Histone H3 PARylation, decreased replication fork speed and defective replication fork restart. An unbiased genome-wide CRISPR screen identified that the loss of HPF1 reverses the SIRT/BRCA synthetic lethality, suggesting serine ADP-ribosylation by PARP1/HPF1 might be toxic to cells that have lost *BRCA*-gene function and SIRT activity.

## Results

### Genetic and small-molecule inhibition of SIRTs is synthetic lethal with *BRCA*-gene defects

**Fig. 1:**
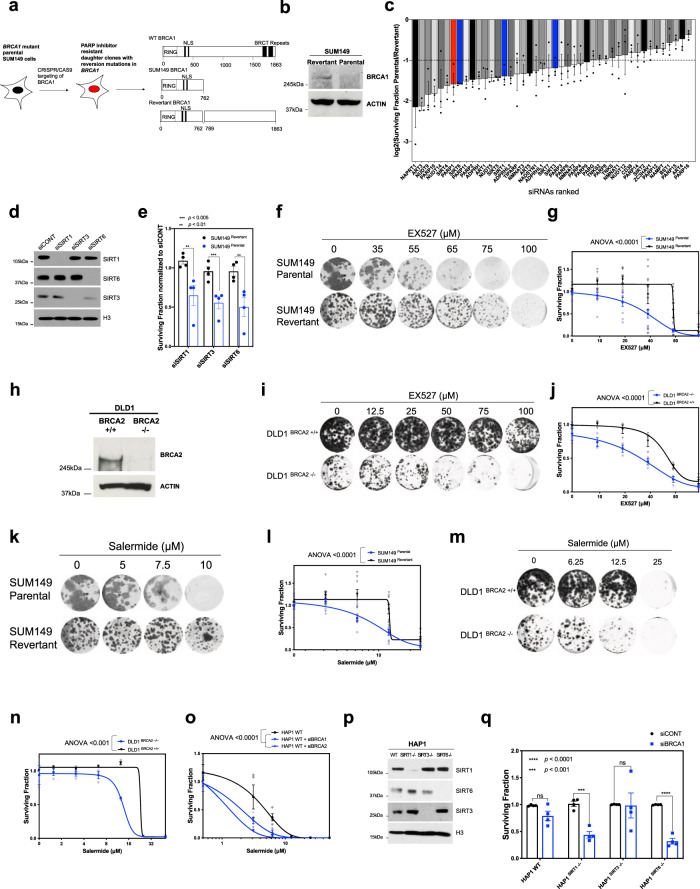
Genetic and small-molecule inhibition of SIRTs is synthetic lethal with BRCA1/2 defects. **a** Schematic showing CRISPR targeting of SUM149 using with Cas9 and CRISPR gRNA expression constructs targeting *BRCA1*, to induce DSB and subsequently create a secondary *BRCA1* mutation reinstating the open reading frame. To the right, predicted BRCA1 protein structure for SUM149 revertant is shown. **b** Western blotting from isogenic *BRCA1* mutant (parental) and *BRCA1* wild-type (revertant) SUM149 cell lysates. Samples were probed with anti-BRCA1 and anti-ACTIN antibodies. **c** Isogenic SUM149 cells transfected with small library of siRNAs targeting NAD^+^ metabolism enzymes. Cells were grown for 7 days and cell viability was then measured using CellTiter-Glo. siCONT normalised surviving fraction in parental SUM149 cells was normalised to that in revertant SUM149 cells. Waterfall plot of log_2_ surviving fractions of SUM149 parental vs. SUM149 revertant cells is shown for each siRNA. Error bars, SEM from three experiments. **d** HEK293T cells were transfected with siRNAs targeting SIRTs 1, 3 and 6. siRNA efficiency was assessed by western blotting, using anti-SIRT1, anti-SIRT3 and anti-SIRT6 antibodies. Anti-H3 was used as a loading control. **e** Isogenic SUM149 cells were transfected with siRNAs targeting SIRTs 1, 3 6. Seven days post transfection, cell viability was assessed using CellTiter-Glo, and values normalised to viability following control siRNA transfection. Data presented as surviving fraction, relative to siRNA control, and analysed using a Student’s *t* test. Error bars, SEM from four independent experiments. **f** Isogenic SUM149 cells were exposed to increasing concentrations of SIRT inhibitor EX527, and grown for 7 days. Cells were then fixed and stained. Representative colony-forming images are shown. **g** Surviving fractions were calculated from cells as in **f**, normalised to DMSO controls and data analysed using an ANOVA with Bonferroni correction for multiple comparisons. Error bars, SEM from nine experiments. **h** Western blotting from isogenic *BRCA2* wild-type (+/+) and *BRCA2* deleted (−/−) DLD1 cell lysates. Samples were probed with anti-BRCA2 and anti-ACTIN antibodies. **i** Isogenic DLD1 cells were exposed to increasing concentrations of SIRT inhibitor EX527, and grown for 7 days. Cells were then fixed and stained. Representative colony-forming images are shown. **j** Surviving fractions were calculated from cells as in **i,** normalised to DMSO controls and data analysed using an ANOVA with a Bonferroni correction for multiple comparisons. Error bars, SEM from three independent experiments. **k** Isogenic SUM149 cells were exposed to increasing concentrations of SIRT inhibitor salermide, and grown for 7 days. Cells were then fixed and stained. Representative colony-forming images are shown. **l** Surviving fractions were calculated from cells as in **k**, normalised to DMSO controls and data analysed using an ANOVA with a Bonferroni correction for multiple comparisons. Error bars, SEM from *n* = 9 independent experiments. **m** Isogenic DLD1 cells were exposed to increasing concentrations of SIRT inhibitor salermide, and grown for 7 days. Cells were then fixed and stained. Representative colony-forming images are shown. **n** Surviving fractions were calculated from cells as in **m**, normalised to DMSO controls and data analysed using an ANOVA with a Bonferroni correction for multiple comparisons. Error bars, SEM from three independent experiments. **o** Wild-type HAP1 cells were transfected with BRCA1 and BRCA2 siRNAs and 24 h later exposed to increasing concentrations of SIRT inhibitor salermide, and grown for 6 days. Surviving fractions were calculated, normalised to DMSO controls and data analysed using an ANOVA with a Bonferroni correction for multiple comparisons. Error bars, SEM from four independent experiments. **p** Wild-type HAP1 cells, alongside *SIRT1*, *SIRT3* and *SIRT6* knockout HAP1 cell lysates were analysed by western blotting using anti-SIRT1, anti-SIRT3 and anti-SIRT6 antibodies. Anti-H3 was used as a loading control. **q** HAP1 isogenic series (WT, SIRT1^–/–^, SIRT3^–/–^, SIRT6^–/–^) were transfected with BRCA1 siRNA or a non-targeting control siCONT siRNA and grown for 6 days. Surviving fractions were calculated, relative to control siRNAs, and analysed using a Student’s *t* test. Error bars, SEM from four independent experiments. Source data are provided in Supplementary Data 6.

In order to understand whether genes involved in NAD^+^ metabolism, other than PARP1, might be synthetic lethal with *BRCA*-gene defects, we carried out parallel siRNA screens in isogenic BRCA1-defective and BRCA1-proficient triple-negative breast cancer cell lines. To do this, we used BRCA1-defective SUM149T (*BRCA1*:c.2288delT (p.N723FsX13), hereafter named SUM149 Parental) and a daughter clone generated by CRISPR mutagenesis, SUM149B1.S^38^ (hereafter named SUM149 Revertant) which had a reversion mutation in *BRCA1*, as well as the parental c.2288delT mutation (c.[2288delT; 2293del80], Fig. 1a). This reversion mutation has previously been shown to restore the native open reading frame of the *BRCA1* c.2288delT allele, encoding a close to full-length 1839 amino acid BRCA1 protein that restores HR, nuclear RAD51 localisation and PARP inhibitor resistance^38^ (Fig. 1a, b). Both cell lines were reverse transfected with a 384 well-plate arrayed siRNA library designed to silence 44 genes associated with NAD^+^ metabolism (a library previously described in^37^). After transfection, cells were continuously cultured for a further 6 days, after which cell viability was estimated by the use of CellTiter-Glo reagent. We carried out three replica screens in each cell line, and then used the CellTiter-Glo luminescence values generated by the replica screens to estimate the differential effects of each siRNA in SUM149 Parental and SUM149 Revertant cells (Fig. 1c).

This approach identified 26 genes that were synthetic lethal with *BRCA1* (*p* < 0.05, Fig. 1c and Supplementary Fig. 1; Supplementary Data 1). Amongst the highly significant hits identified in the siRNA screen were members of the NAD^+^-dependent sirtuin deacetylase family; SIRT1 (Sirtuin 1, SF = 0.36, *p* = 0.002), SIRT3 (Sirtuin 3, SF = 0.41, *p* = 0.03), SIRT4 (Sirtuin 4, SF = 0.34, *p* = 0.006), SIRT5 (Sirtuin 5, SF = 0.38, *p* = 0.007), SIRT6 (Sirtuin 6, SF = 0.33, *p* < 0.0001) and SIRT7 (Sirtuin 7, SF = 0.38, *p* = 0.01). Interestingly, SIRT1 and SIRT6 have recently been implicated in modulating PARP1 activity *via* PARP1 deacetylation or mono-ADP-ribosylation^19,21,22^. SIRT7, as well as regulating RNA polymerase I-mediated transcription, has also been shown to be involved in repair of DSBs by Non-Homologous End Joining by modulating the local accumulation of 53BP1 and DNA-end resection^39^. We also identified mitochondria-associated sirtuins (SIRT3, SIRT4 and SIRT5) as candidate synthetic lethal targets. These genes are involved in the regulation of metabolic enzymes and stress response mechanisms^40,41^.

A number of toolbox small-molecule sirtuin inhibitors exist^42^, including EX527, (SIRT1 IC_50_ = 0.1 μM, SIRT3 IC_50_ = 165 μM, SIRT6 IC_50_ = 107 μM)^43^. We therefore focused our efforts on characterising the synthetic lethal effects of SIRT1, 3 and 6, given their pharmacological tractability. In subsequent validation experiments, we found SIRT1, SIRT3 and SIRT6 gene silencing preferentially inhibited *BRCA1*–deficient SUM149 Parental cells, compared to SUM149 Revertant cells (Fig. 1d, e). EX527 also elicited *BRCA1* synthetic lethality in the SUM149 Parental/Revertant system (Fig. 1f, g, SUM149 Parental SF_50_ = 37 μM, SUM149 Revertant SF_50_ = 66 μM, ANOVA *p* < 0.0001) and *BRCA2* synthetic lethality in BRCA2 isogenic DLD1 cells^44^ (Fig. 1h–j, DLD1 DLD1 *BRCA2*^*–/–*^ SF_50_ = 30 μM*, BRCA2*^*+/+*^ SF_50_ = 57 μM, ANOVA *p* < 0.0001). Synthetic lethality was also reproduced with a different sirtuin inhibitor, salermide^45^ (IC_50_ = 70 μM for SIRT1, in vitro IC_50_ for SIRT3 or SIRT6, not known) (Fig. 1k, l, SUM149 Parental SF_50_ = 6 μM, SUM149 Revertant SF_50_ = 12 μM, ANOVA *p* < 0.0001; Fig. 1m, n, DLD1 *BRCA2*^*–/–*^ SF_50_ = 12 μM, DLD1 *BRCA2*^*+/+*^ SF_50_ = 23 μM, ANOVA *p* < 0.001). MDA-MB-436, a triple-negative breast cancer cell line model harbouring a loss of function mutation in *BRCA1*, also exhibited EX527 and salermide sensitivity (*BRCA1*:c.5396+1G>A, Supplementary Fig. 2a, b, SF_50_ = 18 μM and SF_50_ = 12 μM, respectively), suggesting that this effect was not private to SUM149 or DLD1 isogenic models. Silencing of *BRCA1* in SUM149 revertant cells also caused SIRT inhibitior synthetic lethality, suggesting a causal relationship between *BRCA1* status and SIRT inhibitor synthetic lethality, as it did in non-tumour breast epithelial MCF10A cells (Supplementary Fig. 2c, d). Collectively, our data suggested that both *BRCA1* and *BRCA2* defects are synthetic lethal with SIRT inhibition.

Since small-molecule sirtuin inhibitors have several targets, and since the siRNA screen identified SIRT1, 3 and 6 as candidate *BRCA*-gene synthetic lethal effects, we assessed which sirtuin might be the best candidate to explain the small-molecule inhibitor synthetic lethality (Fig. 1o–q). To assess this, we used HAP1 cells, a near-haploid cell line derived from KBM-7 chronic myeloid leukaemia cells, where either SIRT1, 3 or 6 had been deleted by CRISPR-Cas9 mutagenesis. We first confirmed that in SIRT wild-type cells, *BRCA1* or *BRCA2* silencing caused SIRT inhibitor synthetic lethality (Fig. 1o, siCONT SF_50_ = 5 μM, siBRCA1 SF_50_ = 1 μM, siBRCA2 SF_50_ = 2 μM, ANOVA *p* < 0.0001), confirming that the HAP1 cell line serves as a suitable model to assess these synthetic lethal effects. Next, using HAP1 isogenic models deficient for SIRT1, 3 or 6 (Fig. 1p), we confirmed that SIRT1 and SIRT6 were synthetic lethal with *BRCA1* gene silencing (Fig. 1q, *SIRT1*^*–/–*^ *p* < 0.001, *SIRT6*^*–/–*^ *p* < 0.0001) and that silencing of *BRCA1* in SIRT3 deficient HAP1 cells had negligible effects on cell viability. Taken together, these data suggest that *SIRT1/BRCA1* and *SIRT6/BRCA1* are robust synthetic lethal effects, operating in a range of different cell types. Given the synthetic lethality between PARP inhibitors and *BRCA*-gene defects, we also assessed whether SIRT1 or SIRT6 loss might enhance the cytotoxic effect of PARP inhibitors. Depletion of *BRCA1* caused a similar magnitude of PARP inhibitor sensitivity in SIRT wild-type, *SIRT1*^*–/–*^ or *SIRT6*^*–/–*^ HAP1 cells (Supplementary Fig. 3a–d), suggesting that targeting either of these two Sirtuins would be unlikely to enhance *BRCA1*/PARP inhibitor synthetic lethality.

### SIRT inhibition causes replication fork stress

**Fig. 2:**
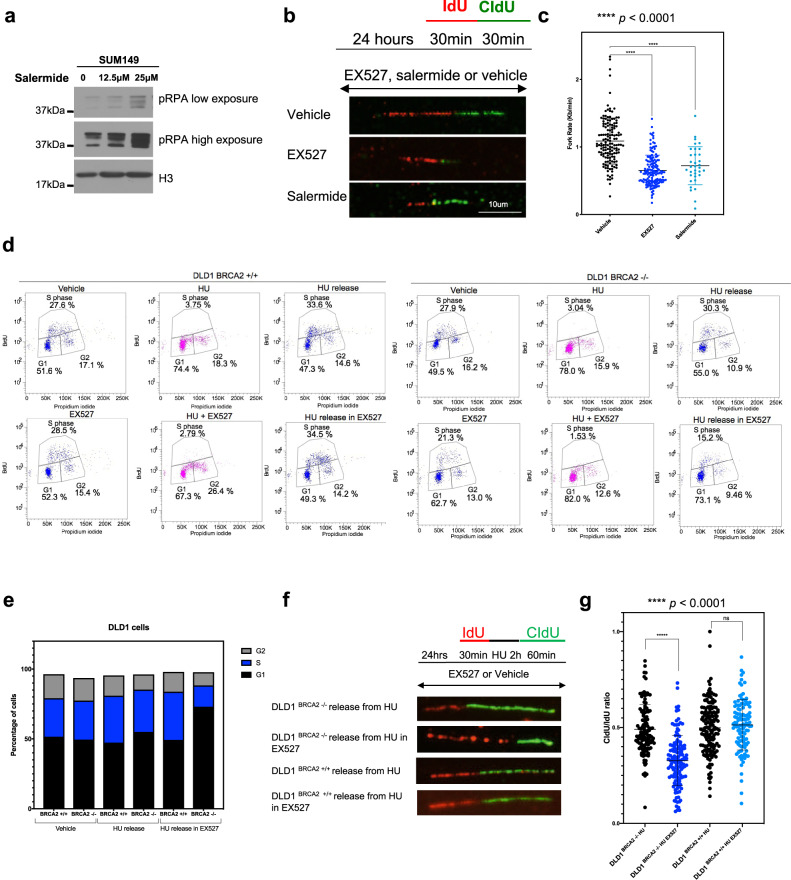
SIRT inhibition causes slower replication fork progression and impaired fork restart in BRCA2-defective cells. **a** SUM149 cells were exposed to vehicle, 12.5 or 25 µM of salermide. After 48 h, chromatin-bound cell lysates were collected and analysed using western blotting, with anti-pRPA and anti-H3 antibodies. **b** DLD1 *BRCA2*^*–/–*^ cells were exposed to 75 µM EX527, 25 µM Salermide or vehicle for 24 h and analysed by DNA fibre assays. Representative images from two biological replicates are shown, scale bar 10 µm. **c** Fork speed was estimated from two biological replicates (*n* = 144 for DLD1 *BRCA2*^*–/–*^ cells exposed to vehicle, *n* = 148 for DLD1 *BRCA2*^*–/–*^ cells exposed to EX527 and n = 34 for DLD1 *BRCA2*^*–/–*^ cells exposed to salermide). Data were assessed using a Student’s *t* test. **d** Isogenic DLD1 cells were pre-treated with EX527 for 24 h, cells were then exposed to 2 mM hydroxyurea for two hours and grown without hydroxyurea for 4 h in media containing 10 µM BrdU. BrdU immunostaining was then performed, alongside propidium iodide, followed by FACS analysis. **e** The percentage of DLD1 *BRCA2*^*+/+*^ or DLD1 *BRCA2*^*–/–*^ cells in G1, active S and G2- phases is shown from figure **d.** Data are representative of two biological replicates. **f** Isogenic DLD1 cells were pre-treated with EX527 or vehicle for 24 h, cells were then exposed to either 2 mM hydroxyurea or DMSO for 2 h and then analysed by DNA fibre assays. Schematic above shows experimental design. Representative images from two biological replicates are shown. **g** CIdU/IdU ratio was estimated from two biological replicates, (*n* = 72 for DLD1 *BRCA2*^*+/+*^ exposed to 2 mM HU, *n* = 112 for DLD1 *BRCA2*^*+/+*^ exposed to 2 mM HU plus EX527, *n* = 128 for DLD1 *BRCA2*^*–/–*^ exposed to 2 mM HU and *n* = 122 for DLD1 *BRCA2*^*–/–*^ exposed to 2 mM HU plus EX527). Data was assessed using a Student’s *t* test. Source data are provided in Supplementary Data 6.

*BRCA1* and *BRCA2* are required for accurate repair and restart of stalled replication forks. In response to replication fork stalling or double-strand break formation at replication forks, H2AX histones in the vicinity of DNA lesions are rapidly phosphorylated, an event that can be monitored by the assessment of discrete, nuclear, γH2AX foci^46^. Despite using concentrations of SIRT inhibitor that elicited *BRCA*-gene synthetic lethality, we did not detect a notable increase in γH2AX foci in either *BRCA*-gene defective or in *BRCA*-gene wild-type cells (Supplementary Fig. 4a, b). A possible explanation for the absence of a γH2AX foci response to SIRT inhibitor might be that SIRT1 inhibition leads to increased H2AX acetylation, which is known to prevent H2AX phosphorylation^47,48^. Consistent with this, silencing *SIRT1* did not cause a profound increase in γH2AX, but silencing *SIRT6* did, in both *BRCA2*-defective and *BRCA2* wild-type cells (Supplementary Fig. 4c, d). It seems reasonable to think that the anti-SIRT1 activity of small-molecule SIRT inhibitors prevents the γH2AX response that might otherwise be caused by their anti-SIRT6 activity. SIRT inhibitor exposure did, however, cause a profound increase in RPA (Replication protein A) phosphorylation, a marker of the single-stranded DNA present at stalled replication forks, suggesting that SIRT inhibitors might be eliciting synthetic lethality by increasing replication fork stress (Fig. 2a). Consistent with this hypothesis, when we carried out a high-throughput RNAi EX527 chemosensitivity screen using a siRNA library targeting 594 DNA damage response genes and genes in the Cancer Gene Census in HR-proficient CAL51 cells (Supplementary Fig. 5, Supplementary Data 2) we found that silencing of genes associated with double-stand break repair *via* HR (adjusted *p* value = 0.0081 (pathway identified by EnrichR^49,50^ pathway analysis), Supplementary Data 3), or those involved in maintaining the integrity of the replication fork, the response of the replication fork to stress and/or the repair of stalled or collapsed replication forks (*ATR*, as well as *RAD1* and *HUS1*, which encode members of the 9-1-1 complex^51^), also sensitised cells to EX527 (adjusted *p* value = 0.0033, Supplementary Data 2 and 3). These data suggest that SIRT inhibitors may have therapeutic potential beyond *BRCA* mutations, and suggest that sirtuins may function to resolve replication stress.

We next used DNA fibre assays to formally assess whether SIRT inhibitors altered replication fork dynamics. Both EX527 or salermide caused a significant reduction in replication fork speed in *BRCA2*-defective cells (Fig. 2b, c). Stalled forks are normally restarted in order to avoid fork collapse and the formation of cytotoxic double-stranded DNA breaks. To assess replication fork restart directly, we analysed the ability of cells to continue DNA replication following release from a 2-h hydroxyurea (HU) exposure. DLD1 *BRCA2*^*+/+*^ or *BRCA2*^–/–^ cells were exposed to SIRT inhibitor for 16 h prior to a 2-h HU replication arrest. After HU was removed, cells were exposed to BrdU and DNA synthesis was monitored by accumulation of BrdU-positive cells. At 0 h after removal of HU, no active DNA synthesis was observed in either *BRCA2*^*+/+*^ or *BRCA2*^–/–^ cells (Fig. 2d). Whilst wild-type cells fully resumed DNA synthesis 4 h after HU release in media containing SIRTi, exposure to EX527 resulted in a significant impairment in replication restart in *BRCA2*^–/–^ cells, where ~15% of cells recovered from HU in the HU plus SIRT inhibitor exposed cells vs. 30% in HU alone (Fig. 2d, e). This defective reactivation of replication in *BRCA2*^–/–^ cells in response to EX527 was also observed with another SIRT inhibitor, salermide (Supplementary Fig. 6a, b), suggesting that these effects were not private to EX527. Consistent with this data, single-molecule analysis of extended DNA fibres extracted from cells exposed to HU plus SIRT inhibitor confirmed abnormalities in replication fork restart in *BRCA2*^–/–^ but not in *BRCA2*^*+/+*^ cells exposed to HU plus SIRT inhibitor (Fig. 2f, g). Moreover, an analysis of IdU-only stained DNA fibres revealed an increase in stalled forks in HU plus SIRT inhibitor exposed *BRCA2*^–/–^ cells when compared to the same treatment in *BRCA2*^*+/+*^cells (Supplementary Fig. 6c). Taken together, our data suggest that in *BRCA*-gene defective cells, sirtuin inhibitors impair replication fork progression and fork restart.

### SIRT inhibitor/*BRCA*-gene synthetic lethality is HPF1 and PARP1 dependent

**Fig. 3:**
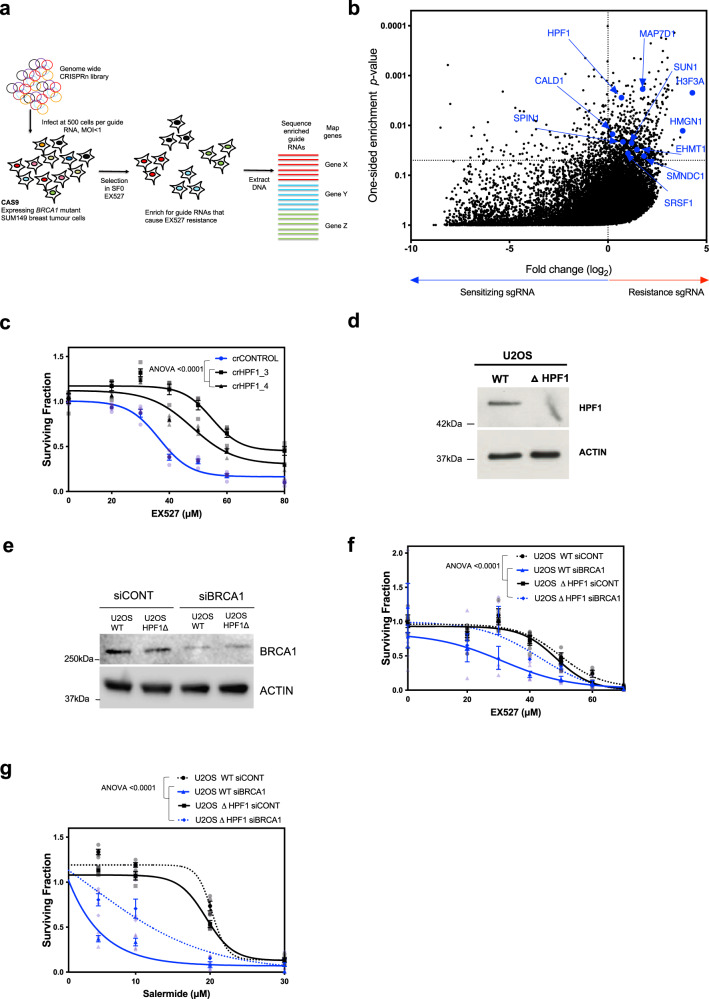
HPF1 loss rescues the SIRT/BRCA SL. **a** Schematic representation of CRISPR screen. Cas9 expressing SUM149 cells infected with a genome-wide CRISPR guide RNA library and grown in an surviving fraction = 0 (SF_0_) concentration of EX527 for 4 weeks. Following enrichment for guides promoting drug resistance, DNA was extracted and the guide enrichment was calculated. **b** Volcano plot illustrating the results from the CRISPR screen. log2 fold change in guide RNA in plasmid vs. EX527 treated SUM149 cells is shown on *x*-axis and *p* values are shown on *y*-axis (log_10_ scale). HPF1 is annotated in blue. **c** SUM149 cells expressing Cas9 were transfected with non-targeting gRNAs or gRNAs targeting HPF1 and were subjected to increasing concentrations of EX527 for 10 days. Cell viability was assessed using CellTiter-Glo, and surviving fractions were calculated, normalised to DMSO, and analysed using an ANOVA with a Bonferroni correction for multiple comparisons. Error bars, SEM from four independent experiments. **d** WT and *HPF1*^*–/–*^ U2OS cell lysates were analysed by western blotting using anti-HPF1 and anti-ACTIN antibodies. **e** Isogenic WT or *HPF1*^*–/–*^ U2OS cell lysates transfected with non-targeting or BRCA1-targeting siRNAs were analysed by western blotting using anti-BRCA1 and anti-ACTIN antibodies. **f** Isogenic U2OS cells were transfected with non-targeting or BRCA1-targeting siRNAs, and were subjected to increasing concentrations of EX527 for 6 days. Cell viability was assessed using CellTiter-Glo, and surviving fractions were calculated, normalised to DMSO, and then analysed using an ANOVA with a Bonferroni correction for multiple comparisons. Error bars, SEM from four experiments. Data are reflective of two biological replicates. **g** Isogenic U2OS cells were transfected with non-targeting or BRCA1-targeting siRNAs, and were subjected to increasing concentrations of salermide for 6 days. Cell viability was assessed using CellTiter-Glo, and surviving fractions were calculated, normalised to DMSO, and then analysed using an ANOVA with a Bonferroni correction for multiple comparisons. Error bars, SEM from four experiments. Data are reflective of two biological replicates. Source data are provided in Supplementary Data 6.

To understand the genetic basis of the sensitivity of *BRCA*-gene defective cells to SIRT inhibition, we used a genome-wide CRISPR-Cas9 mutagenesis (CRISPRn) screen in the *BRCA1* mutant SUM149 Parental cells, to identify genes that when mutated, confer resistance to SIRT inhibition (Fig. 3a). Amongst the significant hits identified that reversed SIRT inhibitor sensitivity were Histone H3.3 and High Mobility Group Nucleosome Binding Domain 1, both of which are targets for serine ADP-ribosylation by PARP1 and its accessory partner, Histone PARylation Factor 1 (HPF1; also known as *C4orf27*) (Fig. 3b and Supplementary Data 4). HPF1 also scored as a hit in the screen (Fig. 3b, *p* = 0.0027). HPF1 is a key regulator of serine ADP-ribosylation in response to DNA damage^7,8^. In addition, we also noted other hits identified in this CRISPR screen were other factors known to be serine ADP-ribosylated by HPF1/PARP1, including *MAP7D1*, *SUN1*, *SPIN1*, *SMNDC1*, *CALD1*, *SRSF1*, *EHMT1* and *ZNF644* (Fig. 3b and Supplementary Data 4). The identification of HPF1 substrates as being associated with SIRT inhibitor resistance provides a mechanistic hypothesis as to how the loss of HPF1 might mediate SIRT inhibitor resistance. As HPF1 is required for serine ADP-ribosylation of several substrates identified in the screen we directly assessed whether the SIRT inhibitor synthetic lethal effect in SUM149 cells was HPF1 dependent and found that two different guide RNAs targeting HPF1 caused SIRT inhibitor resistance in SUM149 cells (Fig. 3c). In *BRCA*-gene wild-type U2OS cells (Fig. 3d, e), silencing of *BRCA1* using previously validated siRNA^52^ caused profound SIRT inhibitor sensitivity (Fig. 3f, g) but this was not seen in isogenic HPF1 knockout cells (Fig. 3f, g). In contrast to the SIRT inhibitor resistance observed in *BRCA1* mutant*/HPF1* defective cells, HPF1 loss caused profound PARP inhibitor sensitivity in both *BRCA*-gene wild-type and *BRCA1*-defective U2OS cells consistent with published literature^8,53^ (Fig. 3e, Supplementary Fig. 7). Taken together, these observations suggested that the SIRT inhibitor/*BRCA*-gene synthetic lethal effect is HPF1 dependent and distinct from PARP inhibitor sensitivity.

Serine ADP-ribosylation is counteracted by the glycohydrolase activity of *ADPRHL2* (ARH3), loss of which has recently been shown to cause PARP inhibitor resistance^53,54^. In the siRNA screen described above, silencing of *ADPRHL2* (ARH3) appeared synthetic lethal in *BRCA1* mutant SUM149 cells^55^, an effect confirmed in validation experiments (Fig. 1c, Supplementary Fig. 1, Supplementary Fig. 8a, b and Supplementary Data 1). This suggested that serine ADP-ribosylation, the process controlled by HPF1, might be essential in *BRCA1* mutant cells and might even explain the SIRT/BRCA synthetic lethality. To test this latter possibility, we assessed whether expression of *ADPRHL2* could reverse the SIRT inhibitor/*BRCA*-gene synthetic lethality. Overexpression of *ADPRHL2* in SUM149 cells caused SIRT inhibitor resistance (Supplementary Fig. 8c, d), suggesting modulators of serine ADP-ribosylation might determine the SIRT inhibitor/*BRCA*-gene synthetic lethality.

**Fig. 4:**
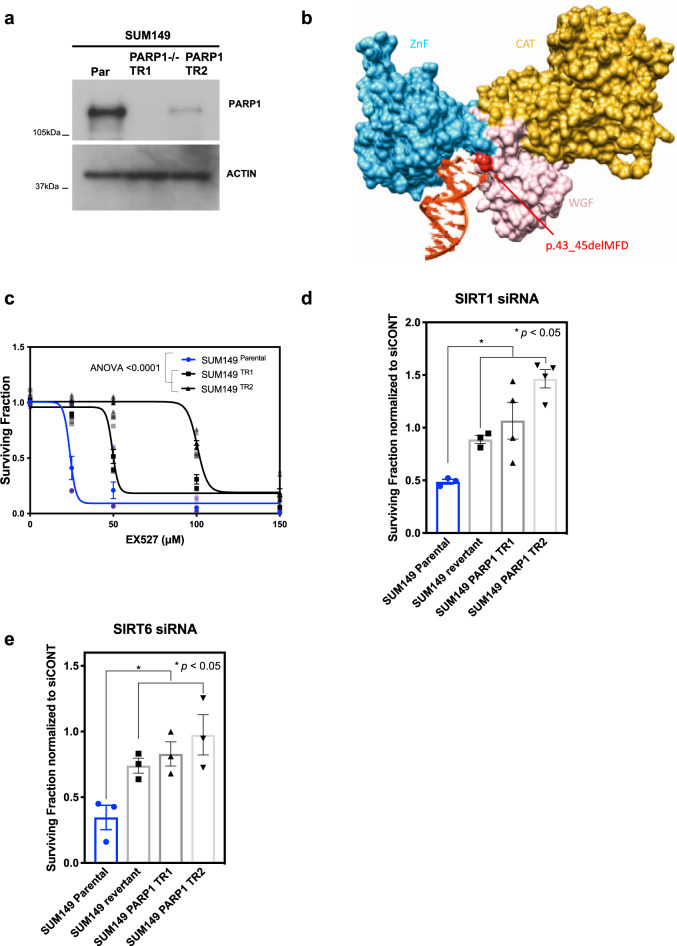
SIRT/BRCA synthetic lethality mediated by SIRT1 and SIRT6 is PARP1 dependent. **a** Western blotting from SUM149 (parental), SUM149 *PARP1* deleted (TR1) and SUM149 *PARP1* ZnF mutant (TR2) cell lysates. Samples were probed with anti-PARP1 and anti-ACTIN antibodies. **b** Schematic representation of *PARP1* TR2 mutant bound to DNA. **c** Isogenic SUM149 cells were exposed to increasing concentrations of SIRT inhibitor EX527, and grown for 7 days. Surviving fractions were calculated, normalised to DMSO controls, and analysed using an ANOVA with a Bonferroni correction for multiple comparisons. Error bars, SEM from n = 11 experiments. **d** Isogenic SUM149 cells were transfected with siRNAs targeting SIRT1. Six days post transfection, cell viability was assessed using CellTiter-Glo, and values normalised to viability following control siRNA transfection. Data presented as surviving fraction, relative to siRNA control. and analysed using a Student’s t-test. Error bars, SEM *n* = 3 for SUM149 parental and revertant and *n* = 4 for SUM149 TR1 and TR4 model. **e** Isogenic SUM149 cells were transfected with siRNAs targeting SIRT6. Six days post transfection, cell viability was assessed using CellTiter-Glo, and values normalised to viability following control siRNA transfection. Data presented as surviving fraction, relative to siRNA control, and analysed using a Student’s *t* test. Error bars, SEM from three independent experiments. Source data are provided in Supplementary Data 6.

HPF1 forms a composite catalytic site with PARP1 to mediate serine ADP-ribosylation^10^, whilst ADPRHL2 counteracts serine ADP-ribosylation^7,8,55^. Given the HPF1 dependency of the SIRT inhibitor synthetic lethality, we assessed whether the SIRT inhibitor/*BRCA*-gene synthetic lethality was PARP1 dependent; to do this we used two previously validated SUM149 subclones that posses either a frameshift mutation in *PARP1* that ablates protein expression^31^ (hereafter named SUM149 TR1) or an in-frame deletion in a zinc finger of the PARP1 DNA-binding domain that prevents DNA binding (hereafter named SUM149 TR2)^31^. Both TR1 and TR2 cells exhibited resistance to SIRT inhibitors (Fig. 4a–c), suggesting that SIRT inhibitor sensitivity was PARP1 dependent and also dependent upon PARP1’s ability to effectively bind DNA. In addition, we found that the *PARP1* mutations in both TR1 and TR2 cells prevented the genetic (as opposed to small-molecule inhibitor elicited) SIRT1 and SIRT6 synthetic lethal effects (Fig. 4d, e). Taken together, these data demonstrated that both the genetic and small-molecule SIRT/*BRCA*-gene synthetic lethal effects were dependent upon wild-type *PARP1* (Fig. 4a–e).

### SIRT inhibitors dysregulate acetylation and PARylation of PARP1 and core histones

**Fig. 5:**
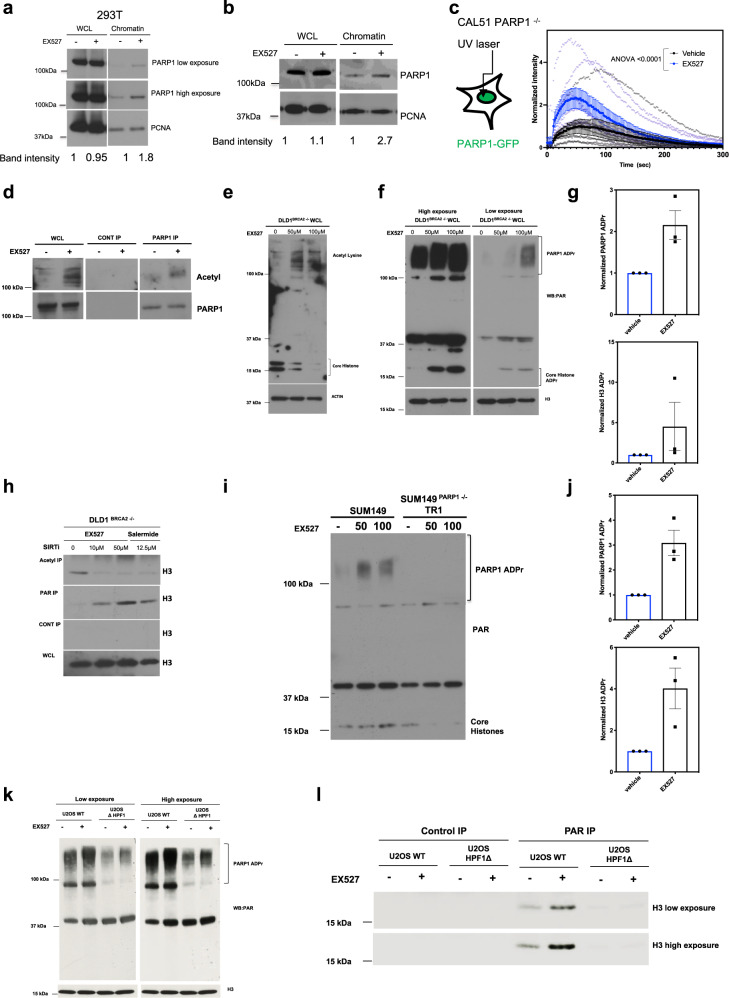
SIRT inhibition leads to increased HPF1-dependent PARylation of PARP1 and H3. **a** HEK293T cells were exposed to 100 µM EX527. After 48 h, whole-cell and chromatin-bound cell lysates were collected and analysed using western blotting, with anti-PARP1 and anti-PCNA antibodies. Data reflective of two biological replicates. **b** Isogenic SUM149 cells were exposed to 75 µM of EX527. After 48 h, whole-cell and chromatin-bound cell lysates were collected and analysed using western blotting, with anti-PARP1 and anti-PCNA antibodies. Data reflective of two biological replicates. **c** To the left, schematic representation of CAL51 cells stably expressing PARP1-GFP. CAL51 cells were laser irradiated in the presence or absence of 100 µM EX527. The recruitment of PARP1 to laser damaged DNA sites was measures and presented as a scatter plot, depicting time on the *x*-axis, and GFP intensity on the *y*-axis. Data representative of eight cells per treatment arm. Data were analysed using an ANOVA with a Bonferroni correction for multiple comparisons. **d** DLD1 *BRCA2*^*–/–*^ cells were immunoprecipitated with anti-PARP1 antibody following a 48 h incubation with 50 µM EX527. Subsequently, precipitates were analysed using western blotting with anti-Acetyl lysine and anti-PARP1 antibodies. **e** DLD1 *BRCA2*^*–/–*^ cells were exposed to 50 or 100 µM EX527 for 48 h. Subsequently, cell lysates were analysed by western blotting using anti-acetyl and anti-H3 antibodies. The first two lanes of the top panel of the anti-Acetyl lysine western blot are also shown in Fig. 5d. **f** DLD1 *BRCA2*^*–/–*^ cells were exposed to 50 or 100 µM EX527 for 48 h. Subsequently, cell lysates were analysed by western blotting using anti-PAR and anti-H3 antibodies. **g** Densitometric quantification of PARylated PARP1 from DLD1 *BRCA2*^*–/–*^ cells exposed to 50 µM EX527 for 48 h from three independent experiments, top, and PARylated H3, bottom, from **f** was normalised to H3 loading control, and presented as fold change over DMSO. All raw western blot data used for the densitometric quantifications are shown in Supplementary Fig. 14. **h** DLD1 *BRCA2*^*–/–*^ cells were immunoprecipitated with anti-acetyl, top, or anti-PAR, bottom, antibodies following a 48 h incubation with EX527 or salermide. Subsequently, immunoprecipitates were analysed using western blotting with anti-H3 antibodies. **i** Parental or *PARP1* SUM149 TR1 cells were exposed to EX527 and subsequently cell lysates were analysed by western blotting using anti-PAR antibodies. **j** Densitometric quantification of PARylated PARP1 from parental SUM149 cells exposed to 100 µM EX527 for 48 h from three independent experiments, left, and PARylated H3, right, from **i** was normalised to ACTIN loading control, and presented as fold-change over DMSO. All raw western blot data used for the densitometric quantifications are shown in Supplementary Fig. 14. **k** Isogenic U2OS cells were exposed to 50 µM of EX527 for 48 h. Subsequently, cell lysates were analysed using western blotting, with anti-PAR and anti-H3 antibodies. **l** Whole-cell lysates as in **k** were immunoprecipitated with anti-PAR antibodies following a 48 h incubation with EX527. Subsequently, precipitates were analysed using western blotting with anti-H3 antibodies. Source data are provided in Supplementary Data 6.

PARP inhibitors, by trapping PARP1 on DNA, cause DNA lesions that are toxic in cells with defective HR^31,32,56^. SIRT1 and SIRT6 modulate PARP1 activity *via* their deacetylase or mono-ADP-ribosylase activities^19,21,22^, which could conceivably alter PARP1 trapping. Given this, and the PARP1 dependency of the SIRT synthetic lethality, we tested whether increased PARP1 trapping could explain the synthetic lethal effects seen. Using a previously described PARP1-chromatin fractionation assay^32^, we found that EX527 only caused a modest enrichment of PARP1 in the chromatin-bound fraction of cell lysates (Fig. 5a, b). Most clinical PARP inhibitors increase the amount of PARP1 bound to DNA but also delay PARP1 dissociation from damaged DNA^31,32^. To test whether SIRT inhibitors also delayed PARP1 dissociation from damaged DNA, we measured the recruitment and dissociation of a PARP1-GFP fusion protein from a microirradiated laser stripe in live cells. We found that EX527 exposure significantly enhanced PARP1 recruitment to sites of damaged DNA (Fig. 5c, Supplementary Fig. 9), as suggested by the PARP1-chromatin immunoprecipitation assay, but in contrast to PARP inhibitor exposure^31^, did not delay PARP1 dissociation from damaged DNA (as measured by the kinetics of GFP signal reduction from the laser stripe). This suggested that although the SIRT inhibitor/*BRCA*-gene synthetic lethality was PARP1 dependent (Fig. 4), it was unlikely to be trivially explained by PARP1 trapping, and thus distinct from the mechanism by which PARP inhibitors elicit synthetic lethality.

Our data until now suggested that the SIRT/*BRCA*-gene synthetic lethal effect was (i) PARP1 dependent; (ii) HPF1 dependent; (iii) reversed by heightened expression of *ADPRHL2*, which counteracts serine ADP-ribosylation; and (iv) was unlikely to be trivially explained by an increase in PARP1 trapping. In order to focus on what could link SIRT inhibition with these phenotypes, we focussed on the role of SIRT1 and SIRT6 in deacetylating PARP1^19,21,22^. For example, increased PARP1 acetylation is a feature of *SIRT1*^*–/–*^ or *SIRT6*^*–/–*^ cells^19,21,22^ and we found that PARP1 acetylation was enhanced by SIRT inhibitor exposure (Fig. 5d). PARP1 auto-PARylation is also coupled to PARP1 acetylation^19^ and again, we found that, consistent with the increase in PARP1 acetylation, PARP1 PARylation was also increased by SIRT inhibitor exposure (Fig. 5e, f). PARP1/HPF1 promotes serine ADP-ribosylation of histones^7,8^ and when this occurs on histone H3 at S10 and S28 residues, this tends to be mutually exclusive with acetylation at adjacent K9 and K27 residues^25^. We found that not only was histone PARylation elevated in cells exposed to SIRT inhibitors, but histone acetylation was decreased (Fig. 5e–g), effects also seen when assessing pan-acetyl and pan-PARylation immunoprecipitates derived from BRCA2-defective cells exposed to SIRT inhibitors (Fig. 5h). In order to confirm that these biochemical events were not exclusive to a single model system, we confirmed that increased PARylation of PARP1 and core histones was also observed in *BRCA1* mutant SUM149 Parental cells, where this modification was found to be PARP1 dependent (Fig. 5i, j). Taken together, our data suggested that SIRT inhibition causes an increase in PARP/HPF1 activity, as demonstrated by the increased PARylation and decreased acetylation of histones. Although we do not as yet know whether the modification of histones is a causative feature of the synthetic lethal effect or merely a consequence of elevated PARP/HPF1 function, the PARP1/HPF1 dependency of the synthetic lethal effect and its ability to be modulated by *ADPRHL2*, suggests that serine ADP-ribosylation might be critical.

**Fig. 6:**
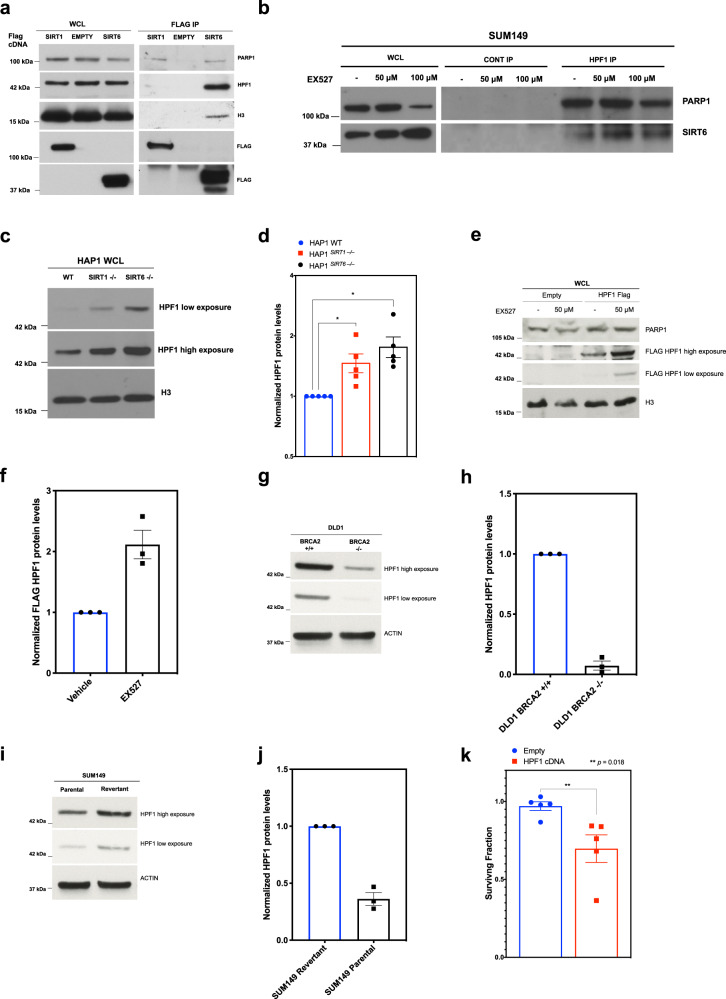
SIRT6 binds to HPF1 and negatively regulates its protein expression. **a** HEK293T cells transfected with a control vector or a vector encoding for SIRT1 or SIRT6 cDNA. 48 h later, whole-cell lysates were then immunoprecipitated with anti-FLAG antibody. Subsequently, immunoprecipitates were analysed using western blotting with anti-PARP1, anti-HPF1, anti-FLAG, and anti-H3 antibodies. **b** SUM149 cells were immunoprecipitated with anti-HPF1, antibody following a 48 h incubation with 50 or 100 µM EX527. Subsequently, immunoprecipitates were analysed using western blotting with anti-PARP1 and anti-SIRT6 antibodies. **c** Wild-type HAP1 cells, alongside *SIRT1* and *SIRT6* knockout HAP1 cell lysates were analysed by western blotting using anti-HPF1 and anti-H3 antibodies. Anti-H3 was used as a loading control. **d** Densitometric quantification of HPF1 from wild-type HAP1 cells, alongside *SIRT1* and *SIRT6* knockout HAP1 cell lysates from five independent experiments, from **c**, were normalised to H3 or ACTIN loading control, and presented as fold-change over wild-type HAP1 cells. All raw western blot data used for the densitometric quantifications are shown in Supplementary Fig. 14. **e** HEK293 cells transfected with FLAG-HPF1 or EMPTY control were exposed to 50 µM EX527 for 48 h incubation. Subsequently, whole cell lysates were analysed by western blotting using anti-FLAG, anti-H3 and anti-PARP1 antibodies. **f** Densitometric quantification of FLAG-HPF1, from **e**. from three independent experiments, normalised to H3 or ACTIN loading control, and presented as fold-change over DMSO. All raw western blot data used for the densitometric quantifications are shown in Supplementary Fig. 14. **g** Western blotting from isogenic *BRCA2* wild-type ^+/+^ and *BRCA2* deleted ^–/–^ DLD1 cell lysates. Samples were probed with anti-HPF1 and anti-ACTIN antibodies. **h** Densitometric quantification of HPF1, from **g**, from three independent experiments, normalised to ACTIN of H3 loading control, and presented as fold-change of *BRCA2* ^–/–^ cells in comparison to *BRCA2* ^+/+^ cells. All raw western blot data used for the densitometric quantifications are shown in Supplementary Fig. 14. **i** Western blotting from isogenic *BRCA1* mutant (parental) and *BRCA1* wild-type (revertant) SUM149 cell lysates. Samples were probed with anti-HPF1 and anti-ACTIN antibodies. **j** Densitometric quantification of HPF1, from **i**, from three independent experiments, normalised to ACTIN or H3 loading control, and presented as fold-change of *BRCA1* mutant (parental) cells in comparison to *BRCA1* wild-type (revertant) SUM149 cells. All raw western blot data used for the densitometric quantifications are shown in Supplementary Fig. 14. **k** SUM149 *BRCA1* mutant (parental) cells were transfected with a control vector or a vector encoding for HPF1 cDNA. Seven days post transfection cell viability was assessed using CellTiter-Glo, and surviving fractions were calculated, normalised to control empty vector. Data were analysed using a Student’s *t* test. Error bars, SEM from five independent experiments. Source data are provided in Supplementary Data 6.

Since HPF1 loss reversed the SIRT/*BRCA*-gene synthetic lethal effect, we wondered whether the increased PARylation associated with SIRT inhibitor exposure was also HPF1 dependent. This was the case; SIRT inhibition increased PARP1 and histone H3 PARylation in cells with HPF1, but these effects were less apparent in HPF1 defective cells (Fig. 5k, l). We also found that the concomitant decrease in core histone acetylation caused by SIRT inhibitor was also PARP1 and HPF1 dependent (Supplementary Fig. 10). To further understand how sirtuins regulate the HPF1/PARP1 complex, we assessed whether SIRT1 and SIRT6 interacted with HPF1/PARP1. Consistent with published data^19,21,22^, flag-tagged SIRT1 or SIRT6 immunoprecipitated with PARP1 (Fig. 6a). SIRT6 also associated with HPF1 and the HPF1/PARP1 target, Histone H3 (Fig. 6a). The association between SIRT6 and HPF1 was also confirmed using immunoprecipitation of the endogenous HPF1 protein and was not affected by SIRT inhibition (Fig. 6b). We also noted from western blotting of wild type, *SIRT1*^*–/–*^ and *SIRT6* ^*–/–*^ HAP1 cells, that HPF1 protein levels were elevated in *SIRT6*^*–/–*^ cells and modestly increased in *SIRT1*^*–/–*^ cells (Fig. 6c, d). Consistent with the genetic depletion of *SIRT1* or *SIRT6*, SIRT inhibitor exposure also caused an increase in HPF1 protein expression (Fig. 6e, f), suggesting that SIRT6 might directly (for example *via* protein interaction) control HPF1 protein levels and activity. We also noted that in isogenic systems, loss of either *BRCA1* or *BRCA2* was associated with a reduction in HPF1 protein levels (Fig. 6g–j); it seems possible that loss of HPF1 expression could represent a homoeostatic response that maintains cell fitness in the face of *BRCA*-gene dysfunction. Consistent with this hypothesis, we found that overexpression of HPF1 had dichotomous effects on cell viability, depending upon the status of *BRCA1*. In cells with functional BRCA1, i.e. Revertant SUM149 cells, HPF1 overexpression promoted cell survival whereas in *BRCA1* defective SUM149 Parental cells, HPF1 overexpression caused a moderate decrease in cell viability (Fig. 6k, Supplementary Fig. 11).

Finally, we assessed the possibility that the synthetic lethal effects we observed were associated with cellular levels of NAD^+^. Increased PARylation (as seen in the SIRT/*BRCA*-gene synthetic lethality) is often associated with NAD^+^ depletion, which in some settings can cause cell death^57^, but can be reversed by experimental supplementation with the NAD^+^ precursor, nicotinic acid (NA)^58,59^. We found that NA rescue did not reverse SIRT genetic or small-molecule inhibitor synthetic lethality, although it did result in: (i) higher levels of cellular NAD^+^; and (ii) reversal of a synthetic lethal effect associated with another NAD^+^ metabolism enzyme, NAMPT which when defective causes synthetic lethality by reducing NAD^+^ levels^37^ (Supplementary Fig. 12a–d). This suggested that NAD^+^ depletion may not be a major cause of SIRT/*BRCA*-gene synthetic lethality.

## Discussion

**Fig. 7:**
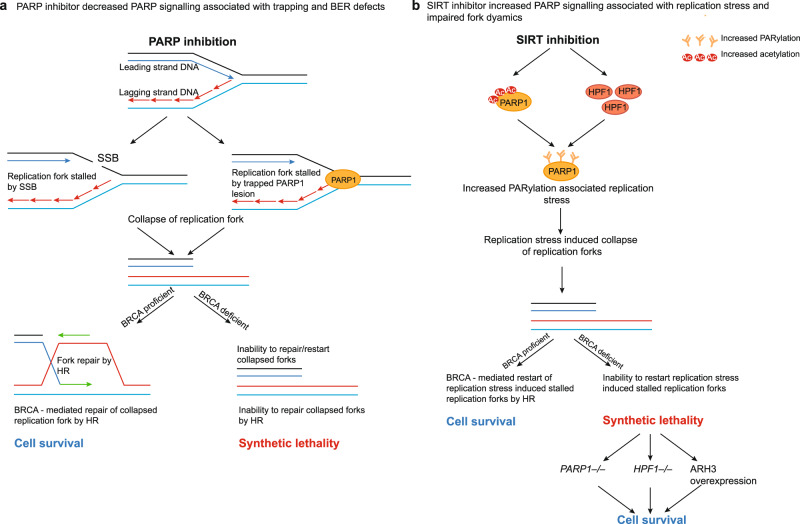
Model of SIRT/*BRCA*-gene synthetic lethality. **a** PARP inhibitors impair SSB repair and also trap PARP1 on DNA, this latter lesion being a major driver of *BRCA*-gene synthetic lethality, as shown. **b** SIRT inhibitors prevent the normal deacetylation of PARP1, leading to increased PARP1/HPF1 activity. SIRT inhibitors also increase replication fork dysfunction in *BRCA*-gene mutant cells, a likely driver of the synthetic lethality. Genetic deletion of *PARP1* or *HPF1* reverses the synthetic lethal effect of SIRT inhibitors, as does ectopic expression of ARH3 a PAR hydrolase, which normally degrades serine ADP-ribosylated chains; taken together these observations implicate the serine ADP-ribosylation activity of PARP1/HPF1 as being a causative feature of this synthetic lethality.

*BRCA1* or *BRCA2* are recurrently mutated in cancers of the breast, ovary, pancreas or prostate. Despite drugs such as platinum salts or PARP inhibitors providing therapeutic options for treating these diseases, these drugs are not effective in all *BRCA*-gene mutant patients, suggesting alternative approaches to synthetic lethal targeting of these genes is critical. Here, we show that either genetic or chemical inhibition of sirtuin enzymes is synthetically lethal with *BRCA*-gene defects and is characterised by increased replication fork stress. Analogous to the PARP/BRCA synthetic lethality, the SIRT/BRCA synthetic lethality is PARP1 dependent. Unlike PARP inhibitor synthetic lethality, however, inhibiting sirtuins leads to increased PARP1 activity, characterised by increased PARP1 acetylation, increased PARP1 auto-PARylation and increased PARylation of the PARP1 substrate, histone H3. While we cannot as yet confirm whether all of these observations represent causative features of the synthetic lethality, the HPF1 and PARP1 dependency of the SIRT/*BRCA*-gene synthetic lethality and its ability to be reversed by overexpression of ARH3 (*ADPRHL2*) suggests that the shared function of these proteins, namely the control of serine ADP-ribosylation, might be critical (Fig. 7).

What substrate or indeed substrates of serine ADP-ribosylation are key to the SIRT/*BRCA*-gene synthetic lethality remain unknown as yet. It is also not clear what the critical threshold of serine ADP-ribosylation and PARylation might be that determines the switch from *BRCA*-gene mutant cell fitness to synthetic lethality. Nevertheless, cytotoxicity associated with excessive PARylation (for example as occurs in parthanatos) has some precedence in cancer and other diseases^54,60–62^, including the recent demonstration that PARP1-hyperactivity causes neuronal cell loss associated with cerebellar ataxia^57^. In this latter case, increased PARP1 activity is cytotoxic in neuronal cells as it leads to excessive depletion of NAD^+^ levels^57^. Here, we were not able to reverse the SIRT/*BRCA*-gene synthetic lethality by supplementation of cells with the NAD^+^ precursor, NA. Thus, we reason that the SIRT/BRCA-gene synthetic lethality is unlikely to be a consequence of NAD^+^ exhaustion, and more likely to be a consequence of aberrant DNA damage response signalling.

In addition, recent work has shown that the loss of ARH3, which is the main hydrolase of endogenous serine-linked mono-ADP-ribosylation, and PARG, which removes long PAR chains, are highly toxic to cells due to unrestrained PARylation^54^. This is consistent with our data whereby increased serine ADP ribosylation induced by HPF1/PARP1 or overexpression of ARH3 in response to SIRT inhibition is toxic to *BRCA* defective cells which have increased endogenous levels of PARylation. Similar to our data in response to SIRT inhibition, excessive accumulation of PARylation upon simultaneous loss of ARH3 and PARG activity leads to a substantial decrease in histone acetylation and as a consequence dysregulated transcription^54^. Whilst we did not assess the effects on transcription in SIRT inhibitor exposed cells, it is possible that the toxicity observed in *BRCA* mutant cells could also be linked to dysregulated transcription.

Interestingly, human *BRCA1*-associated breast cancers have been shown to have lower levels of SIRT1 than their normal controls (Supplementary Fig. 13), an effect likely caused *via* the transcriptional effects of BRCA1 on *SIRT1* expression^63,64^. It is possible that these lower levels of SIRT1 in *BRCA1* mutant cells exacerbate the sensitivity to SIRT1/6 knockdown or inhibition that we have described here. Our results also suggest that there is an interplay between SIRT and HR defects more generally, as we have also observed synthetic lethality with both *BRCA1* and *BRCA2* which is not known to have direct transcriptional effects on *SIRT* genes.

In summary, we identify a previously uncharacterised synthetic lethal relationship between SIRT/BRCA. The events that are associated with SIRT inhibitor/*BRCA*-gene synthetic lethality include: (1) reduced replication fork speed; (2) impaired fork restart; (3) increased PARylation; (4) PARP1 and HPF1 dependency (Fig. 7). The finding that loss of PARP1/HPF1 or gain of *ADPRHL2* rescues the SIRT/BRCA synthetic lethality, suggests an unexpected toxic molecular event linked to serine ADP-ribosylation that selectively targets *BRCA*-gene defective cells.

## Methods

### Materials

The EX527/Salermide (Selleck Chemicals) were used as previously described^65,66^. NA, HU and BrdU, IdU and CIdU were all purchased from Sigma-Aldrich and used as described previously^37,67^.

### Cell culture

DLD1 BRCA2^+/+^ and ^–/–^ (Horizon Discovery), HAP1 WT, SIRT1^–/–^, SIRT3^–/–^, SIRT6^–/–^ (Horizon Discovery), CAL51 (DSMZ), HEK293T, MDA-MB-436 (ATCC) and U2OS WT and U2OS HPF1^–/–^ (gift from Ivan Ahel) cells were maintained in Dulbecco’s modified Eagle’s medium (DMEM) supplemented with 10% FBS (with 10 µg/ml insulin in the case of MDA-MB-436). SUM149 cells (Asterand Bioscience) were maintained in Ham’s F-12 medium supplemented with 5% FBS, 10 µg/ml insulin and 1 µg/ml hydrocortisone.

All human cell line identities were confirmed by STR typing and verified free of mycoplasma infection using Lonza MycoAlert.

### RNAi screening

Cell lines were transfected with SMARTpool siRNAs, using Lipofectamine 2000 (Invitrogen) transfection reagent. The siRNA library (siARRAY—targeting 44 known and putative human PARPs and proteins with an established role in NAD^+^ metabolism) was purchased from Dharmacon. Each well in this library contained a SMARTpool of four distinct siRNA species targeting different sequences of the target transcript. Each plate was supplemented with siCONTROL (10 wells; Dharmacon). Cells were cultured for 6 days after transfection, at which point cell viability was estimated by the use of CellTiter-Glo (Promega). Luminescence readings from each 384 well were normalised to the median of siCONT for each plate for each cell line. The siCONT normalised surviving fractions (SFs) in parental SUM149 cells were then normalised to that in revertant SUM149 cells. For the high-throughput RNAi EX527 drug sensitivity screen a library of 594 DNA damage response (DDR) genes and genes in the Cancer Gene Census (CGC) (Supplementary Data 5) were used to transfect CAL51 cells. Forty-eight hours post-transfection cells were exposed to 50 μM EX527. Cells were cultured for 6 days after transfection, at which point cell viability was estimated by use of CellTiter-Glo (Promega). Luminescence readings from each 384 well were log_2_ transformed, centred according to plate median effects and then *Z*-score standardised according to the library median effect and the median absolute deviation. In total we used data from three replicates in the final analysis.

### Genome-wide CRISPR screen in SUM149 cells

SUM149-Cas9 cells were generated by transduction of SUM149 cells with a Cas9-bsd lentivirus and selection in 7 µg/ml blasticidin. Cells were infected at multiplicity of infection (MOI) 0.3 with a previously published genome-wide human lentiviral CRISPR library^68^. Cells were selected with puromycin and then placed under EX527 selection at a concentration that killed all non-infected SUM149 cells (100 μM). sgRNA sequences from resistant cells were PCR amplified and sequenced.

### Analysis of genome-wide CRISPR screens

After PCR amplification and sequencing, coverage-normalised read counts for the surviving population were compared to read counts from the plasmid population used to prepare the lentiviral CRISPR library. Screens were analysed using the Mageck robust rank aggregation algorithm^69^ to generate genewise fold change and *p* value for enrichment in the surviving population.

### Cell survival assays

Cell survival assays were performed in 96‐well plates. For measurement of sensitivity to EX527 and Salermide, cells were seeded in 96‐well plates at a concentration of 200–750 cells per well. Twenty‐four hours post‐seeding, drug treatment was initiated and cells were continuously exposed to the drug with media. After 7 days, cell viability was estimated using CellTiter-Glo (Promega). SFs were calculated and drug sensitivity curves plotted as previously described^70^.

### Protein analysis

Whole‐cell protein extracts were prepared from cells lysed in NP250 buffer (20 mM Tris pH 7.6, 1 mM EDTA, 0.5% NP40, 250 mM NaCl); supplemented with protease inhibitor cocktail tablets (Roche, Burgess Hill, UK). Protein concentrations were measured using BioRad Protein Assay Reagent (BioRad, Hemel Hempstead, UK). For Western blot analysis, 50 µg of whole-cell lysates were electrophoresed on Novex 4–12% gradient *bis*–*tris* pre‐cast gels (Invitrogen) and immunoblotted overnight at 4 °C with antibodies listed in Supplementary Table 1. Incubation with primary antibody was followed by incubation with a horseradish peroxidase‐conjugated secondary antibody and chemiluminescent detection of proteins (Amersham Pharmacia, Cardiff, UK). Immunoprecipitations were performed by incubating protein G Dynabeads (Sigma) bound by the anti-Acetyl or anti-PAR (Trevigen) antibody or Anti-FLAG M2 magnetic beads (Sigma-Aldrich) at 4 °C overnight. Beads were then washed three times in 1 ml cold NP250 buffer (Tris pH 7.6, 1 mM EDTA, 0.5% NP40, 250 mM NaCl) and eluted using Laemlli SDS sample buffer diluted in lysis buffer. Immunoprecipitated proteins were then electrophoresed on a Novex 4–12% gradient *bis*–*tris* pre‐cast gels (Invitrogen) and immunoblotted overnight at 4 °C with antibodies listed in Supplementary Table 1.

### NAD/NADH assay

For measurement of cellular NAD^+^ levels, 250,000 cells were plated into six‐well plates and 24 h later drug treatment was initiated. 48 h later cells were processed using the NAD/NADH kit (Abcam) according to the manufacturer’s instructions.

### Plasmids and CRISPR/Cas9 crRNA sequences

Human HPF1, ADPRHL2, SIRT1 and SIRT6 expression constructs, in pCMV6-entry and pCMV6-entry empty vector were obtained from Origene. The following guide RNA (gRNA) target sequences were used in this study: for C4orf27-crRNA 3, 5′-GATGAATTTCCTGTATATGT-3′ (exon 4); and for C4orf27-crRNA 4, 5′-AACCAAGCCTGCACCATGAA-3′ (exon 6).

### Chromatin extraction

Chromatin lysates were prepared using a protocol described previously^71^. Cytoplasmic lysate was removed by lysing cells in hypotonic buffer for 10 min, and nuclear fractions were then collected by centrifugation at 4000 × *g* for 3 min at 4 °C. Next, nuclear membranes were disrupted by re-suspending cells in hypertonic buffer for 20–30 min and centrifuged at full-speed for 10 min at 4 °C. The supernatant, containing nuclear soluble proteins, was discarded. The remaining pellet, containing the chromatin fraction, was washed once with hypertonic buffer (to remove residual nuclear soluble proteins), re-centrifuged, and re-suspended in NP250. Chromatin was then sheared by sonication, and insoluble proteins removed by centrifugation at full speed for 10 min at 4 °C.

### Microirradiation assays

CAL51 cells with stable PARP1-GFP were grown in glass-bottom culture dishes (MaTek, P35G-0.170-14-C) in 10% FBS DMEM media and maintained at 37 °C and 5% CO_2_ in an incubation chamber mounted on the microscope. Cells were preincubated with 75 μM EX527 prior to imaging. Imaging was carried out on Andor Revolution system, 60x water objective with micropoint at 365 nm. Only cells with similar GFP signal intensity were measured. The background intensity (in the vicinity of the microirradiation area in the nucleus) was subtracted from that at the microirradiation point and the maximum was normalised to 1.

### Immunocytochemistry

Immunocytochemistry was performed using a protocol described previously^71^. Cells were washed 3× with PBS, fixed in 4% PFA for 10 min, and washed 3× with PBS. Subsequently, cells were permeablised using 0.5% Triton-X in PBS for 5 min and incubated with 3% BSA for 20 min. Cells were then incubated with primary γH2AX antibodies (JBW301) in 3% BSA overnight at 4 °C. Cells were then washed 3× with PBS, incubated with Alexa Fluor-555 conjugated secondary antibody and DAPI for 1 h at room temperature, and were then washed a further 3× with PBS, before being imaged.

High content imaging was performed in the wells of a 96-well plate using the image express (Molecular Devices) platform, using a 40× lens. Non-biased foci analysis was performed using the metaxpress software, which allows for threshold-based quantification of nuclear foci. At least 1000 cells were counted per condition, per biological replica.

### DNA fibre analysis

DLD1 BRCA2^+/+^ and ^−/−^ were plated into six-well plates and exposed to 75 μM of EX527 for 16 h after which they were pulse labelled with IdU/CIdU for 30 min each. To assess fork restart, isogenic DLD1 cells were pre-treated with EX527 or vehicle for 24 h, cells were then exposed to either 2 mM HU or DMSO for 2 h and then analysed by DNA fibre assays. DNA fibre assays were then carried out as described previously^67,72^.

### FACS analysis

Cells were plated in six-well plates and exposed to either 75 μM EX527, 25 μM Salermide or DMSO. Cells were pulse labelled with 10 μM BrdU for 60 min prior to fixation in ice-cold 70% (v/v) ethanol and stored at −20 °C until use. BrdU was fluorescently labelled using an anti BrdU antibody for 1 h at room temperature followed by a secondary Alexa Fluor 488 fluorophore antibody^67^. Total DNA content was assessed by incubation of cells for 30 min in propidium iodide (PI, Sigma-Aldrich). Cell-cycle profiles were generated using a BD LSR-II flow cytometer (BD Biosciences) and analysis was performed using FlowJo software V8.01. Flow cytometry was performed according to the manufacturer’s guidelines.

### Statistics and reproducibility

Data from the siRNA screen was processed as described in the main text and also in ref. ^52^. Data from comparative groups in the in vitro drug sensitivity assays were compared using ANOVA (either two way or two-way repeated measures, as appropriate) in the Graphpad Prism software package or Student’s *t* test where appropriate. All experiments were performed in at least biological triplicate with similar results. Where only two biological repeats were performed, this is noted in the figure legends. The standard error of the mean (SEM) were calculated based on at least three biological experiments.

### Reporting summary

Further information on research design is available in the Nature Research Reporting Summary linked to this article.

## Supplementary information

Supplementary Information

Description of Additional Supplementary Files

Supplementary Data 1

Supplementary Data 2

Supplementary Data 3

Supplementary Data 4

Supplementary Data 5

Supplementary Data 6

Supplementary Data 7

Reporting Summary

## Data Availability

All relevant data presented in this manuscript are included in the paper and in the supplementary information files. Original western blot data are provided as Supplementary Fig. 14 and source data files are provided in Supplementary Data 6 and 7. All datasets generated during and/or analysed during the current study are available from the corresponding author on reasonable request.
