# Supplementary material for: Sirtuin inhibition is synthetic lethal with BRCA1 or BRCA2 deficiency
Source: Commun Biol. 2021 Nov 8;4:1270. doi: 10.1038/s42003-021-02770-2 (PMC8575930; doi:10.1038/s42003-021-02770-2)
Supplement: Supplementary file 1 — Supplementary Information [file 42003_2021_2770_MOESM1_ESM.pdf]

Sirtuin inhibition is synthetic lethal with *BRCA1* or *BRCA2* deficiency

Ilirjana Bajrami[1,2,3]*, Callum Walker[2]*, Dragomir B. Krastev[1,2], Daniel Weekes[2], Feifei Song[1,2], Andrew Wicks[1,2], John Alexander[2], Syed Haider[2], Rachel Brough[1,2], Stephen J. Pettitt[1,2,4], Andrew N.J. Tutt[2,4] and Christopher J. Lord[1,2,4]

Supplementary Table 1 – List of antibodies used

Antibody	Catalogue Number	Company	Dilution
BRCA1	OP92	Calbiochem	1/200
BRCA2	OP95	Calbiochem	1/500
ACTIN	ab8226	Abcam	1/1000
FLAG	F3165	Sigma	1/1000
PCNA	ab18197-100	Abcam	1/1000
Histone H3	ab1791	Abcam	1/1000
γH2AX (S139) (JBW301)	05-636	Millipore	1/1000
PAR Polymer	4335-MC-100	Trevigen	1/1000
PARP1	9542	Cell signalling	1/1000
SIRT1	8469S	Cell signalling	1/1000
SIRT3	2627S	Cell signalling	1/1000
SIRT6	12486S	Cell signalling	1/1000
pRPA32 (S4/S8)	A300-245A	Bethyl	1/1000
HPF1	NBP1-93973	NovusBiologicals	1/1000
Acetylated-Lysine	9441S	Cell signalling	1/1000
BrDU	347580	BD Biosciences	1/100

Supplementary Fig. 1

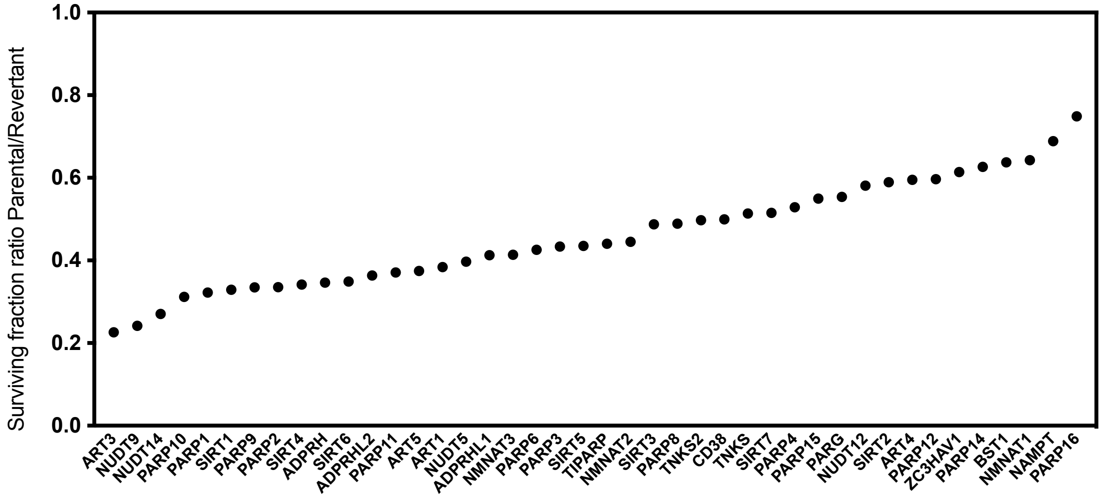

Supplementary Fig. 1. Genetic inhibition of SIRTs is synthetic lethal with BRCA1/2 defects. Isogenic SUM149 cells transfected with small library of siRNAs targeting NAD^+ metabolism enzymes. Cells were grown for seven days and cell viability was then measured using CellTiter-Glo. Surviving fraction in parental SUM149 cells was normalised to that in revertant SUM149 cells. Heatmap representation of surviving fraction ratios of SUM149 parental vs SUM149 revertant cells are shown for each siRNA. Data reflective of three replicates. Source data are provided in Supplementary Data 8.

Supplementary Fig. 2

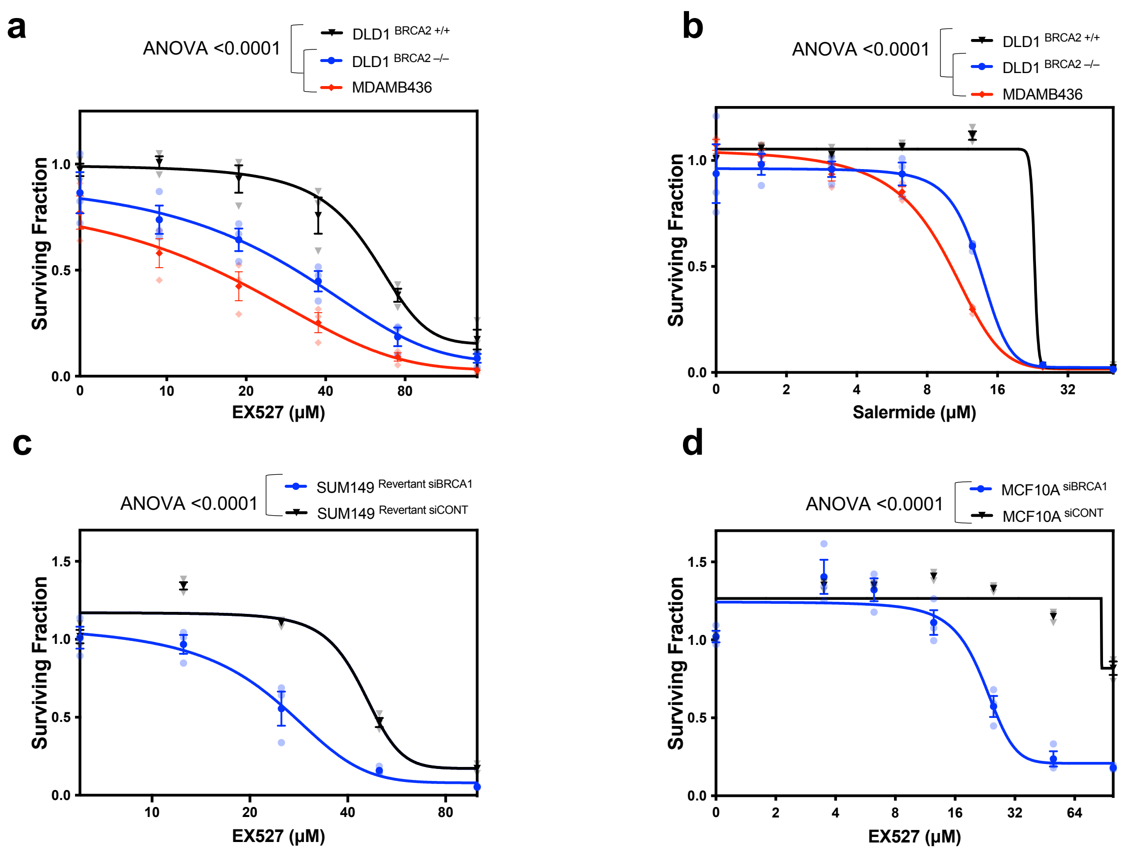

Supplementary Fig. 2. SIRT inhibition is selective with *BRCA*-gene defects. a Isogenic DLD1 cells from **Fig. 1j** and MDAMB436 *BRCA1* mutant breast tumour cells were exposed to increasing concentrations of SIRT inhibitor EX527, and grown for six days. Surviving fractions were calculated, normalised to DMSO controls and analysed using an ANOVA with a Bonferroni correction for multiple comparisons. Error bars, SEM from three independent experiments. **b** Isogenic DLD1 cells from **Fig. 1n** and MDAMB436 *BRCA1* mutant breast tumour cells were exposed to increasing concentrations of SIRT inhibitor salermide, and grown for six days. Surviving fractions were calculated, normalised to DMSO controls and analysed using an ANOVA with a Bonferroni correction for multiple comparisons. Error bars, SEM from three independent experiments. **c** SUM149 *BRCA1* (revertant) cells were transfected with non-targeting or BRCA1-targeting siRNAs, and were subjected to increasing concentrations of EX527 for six days. Cell viability was assessed using CellTiter-Glo, and surviving fractions were calculated, normalised to DMSO, and then analysed using an ANOVA with a Bonferroni correction for multiple comparisons. Error bars, SEM from three independent experiments. **d** MCF10A cells were transfected with non-targeting or BRCA1-targeting siRNAs, and were subjected to increasing concentrations of EX527 for six days. Cell viability was assessed using CellTiter-Glo, and surviving fractions were calculated, normalised to DMSO, and then analysed using an ANOVA with a Bonferroni correction for multiple comparisons. Error bars, SEM from three independent experiments. Source data are provided in Supplementary Data 8.

Supplementary Fig. 3

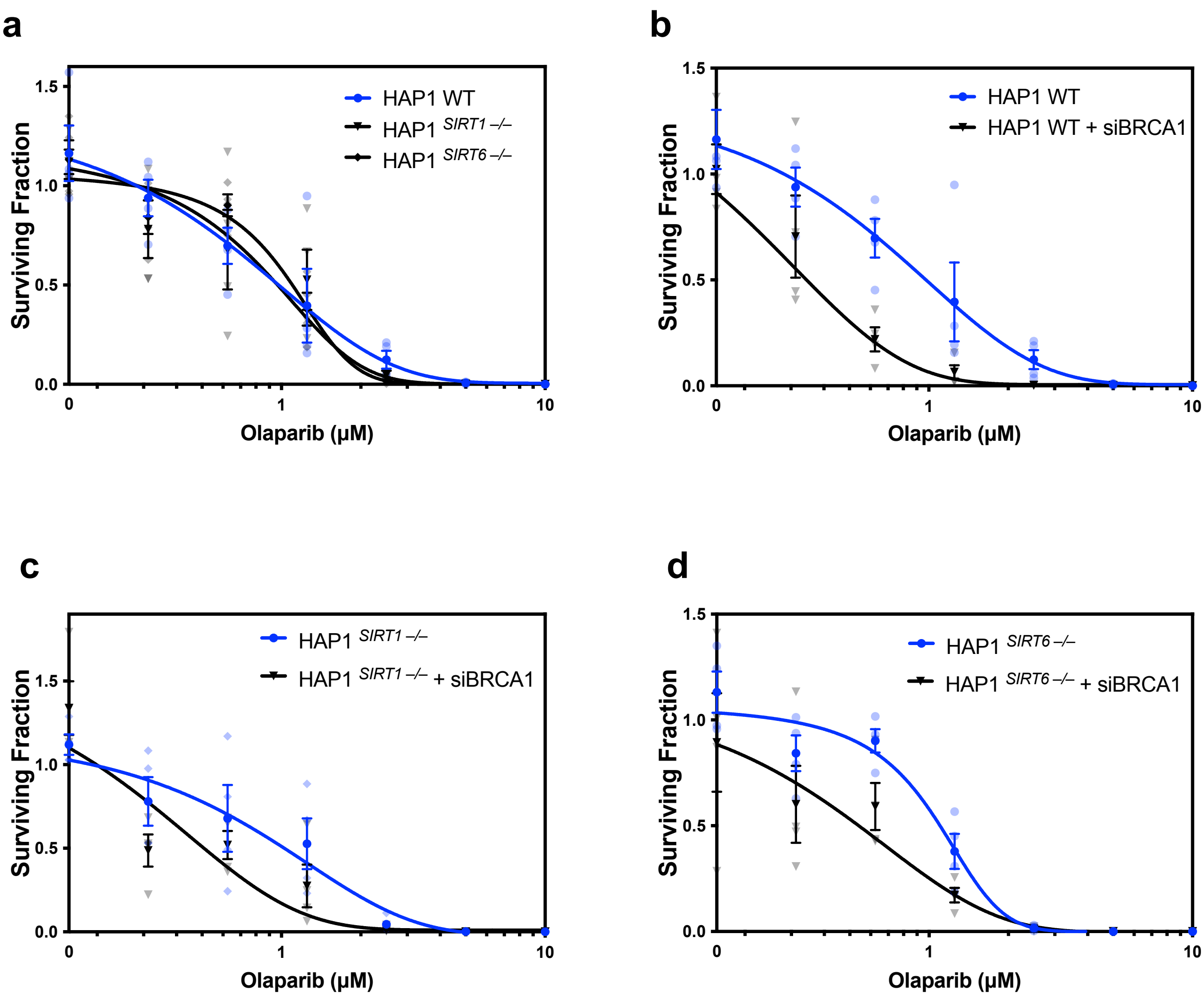

Supplementary Fig. 3. PARP inhibitor sensitivity in *SIRT1* and *SIRT6* deficient cells. a Wild-type HAP1, alongside *SIRT1* and *SIRT6* knockout HAP1 cells exposed to increasing concentrations of PARP inhibitor olaparib, and grown for six days. Surviving fractions were calculated, normalised to DMSO controls and data analysed using an ANOVA with a Bonferroni correction for multiple comparisons. Error bars, SEM from n = 4 experiments. **b** Wild-type HAP1 **c** SIRT1 knockout HAP1 **d** SIRT6 knockout HAP1 cells were transfected with siCONT or BRCA1 siRNAs, and 24 hours later exposed to increasing concentrations of PARP inhibitor olaparib, and grown for six days. Surviving fractions were calculated, normalised to DMSO controls and data analysed using an ANOVA with a Bonferroni correction for multiple comparisons. Error bars, SEM from n = 4 experiments. Source data are provided in Supplementary Data 8.

Supplementary Fig. 4

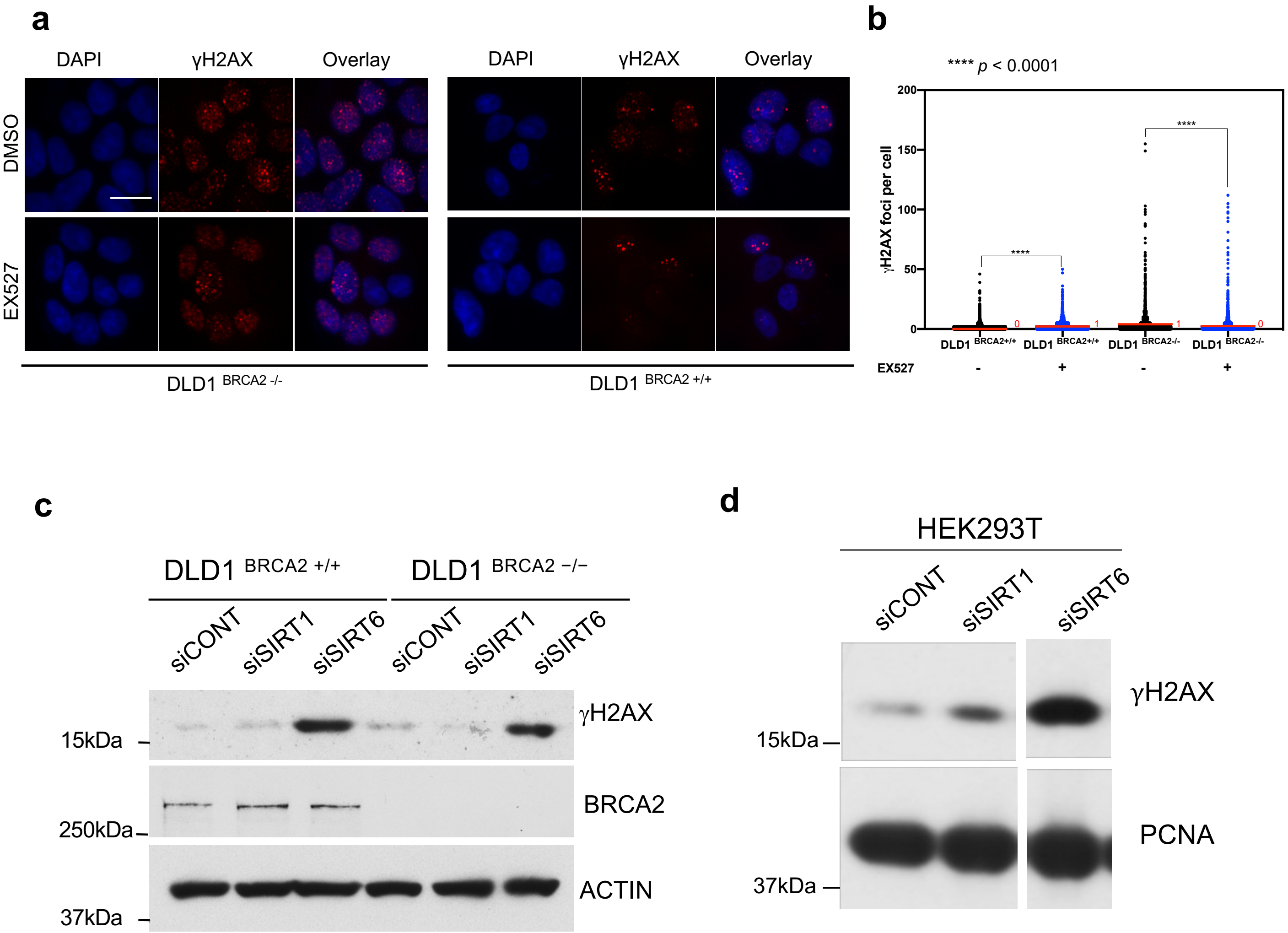

Supplementary Fig. 4. γH2AX response to SIRT inhibition in BRCA2 defective isogenic cells. a Isogenic DLD1 cells were exposed to 50 μM EX527 for 48 hours and analysed by immunocytochemistry using anti-γH2AX antibodies. Representative images are shown from 2 biological replicates, scale bar 10 μm. **b** Isogenic DLD1 cells were exposed to 50 μM EX527 for 48 hours and analysed by immunocytochemistry using anti-γH2AX antibodies. The number of γH2AX foci per cell was quantified using high-throughput confocal microscopy. Data presented is from 2 biological replicates, >1000 cells total. Data was analysed using a Student's t-test with Bonferroni correction for multiple comparisons. **c** Isogenic *BRCA2* wild-type ($^{+/+}$) and *BRCA2* deleted ($^{-/-}$) DLD1 cells were transfected with siCONT, alongside siSIRT1 and siSIRT6 siRNA. Subsequently, whole cell lysates were analysed by western blotting using anti-γH2AX, anti-BRCA2 and anti-ACTIN antibodies. Anti-ACTIN was used as a loading control. **d** HEK293T cells were transfected with siCONT, alongside siSIRT1 and siSIRT6 siRNA. Subsequently, whole cell lysates were analysed by western blotting using anti-γH2AX and anti-PCNA antibodies. Anti-PCNA was used as a loading control. Source data are provided in Supplementary Data 8.

Supplementary Fig. 5

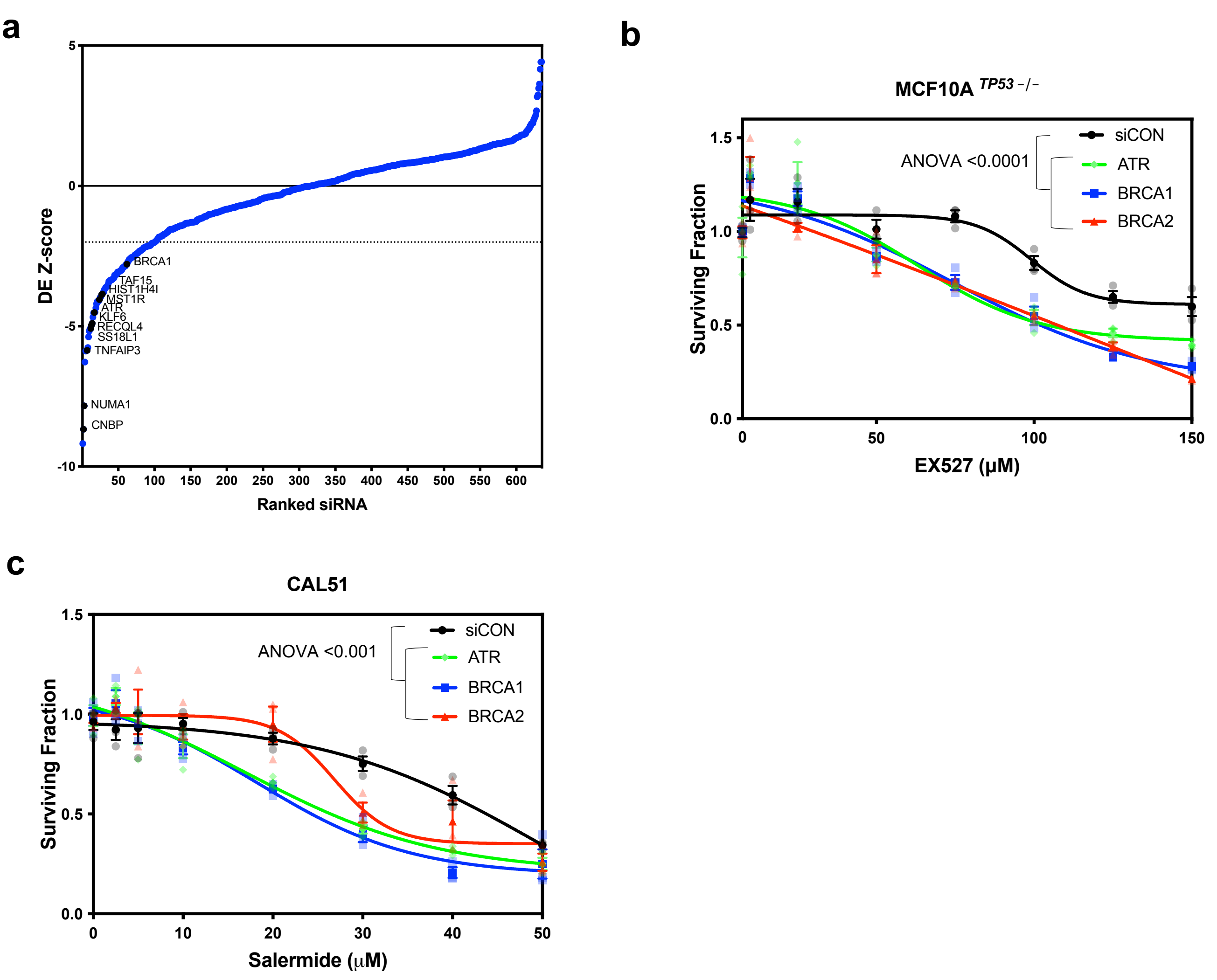

Supplementary Fig. 5. SIRT inhibitor screen of DDR and CGC genes identifies additional HR factors and factors involved in replication fork dynamics. a Plot of median EX527 DE Z scores from 594 siRNA SMARTpools used in the chemosensitization screens. Each siRNA is ranked by its median DE Z score. **b** MCF10A cells were transfected with BRCA1, BRCA2, ATR or a non targeting control siRNA, and 24 hours later exposed to increasing concentrations of SIRT inhibitor EX527, and grown for six days. Surviving fractions were calculated, normalised to DMSO controls and analysed using an ANOVA with a Bonferroni correction for multiple comparisons. Error bars, SEM from three independent experiments. **c** CAL51 cells were transfected with BRCA1, BRCA2, ATR or a non targeting control siRNA, and 24 hours later exposed to increasing concentrations of SIRT inhibitor salermide, and grown for six days. Surviving fractions were calculated, normalised to DMSO controls and analysed using an ANOVA with a Bonferroni correction for multiple comparisons. Error bars, SEM from three independent experiments. Source data are provided in Supplementary Data 8.

Supplementary Fig. 6

a

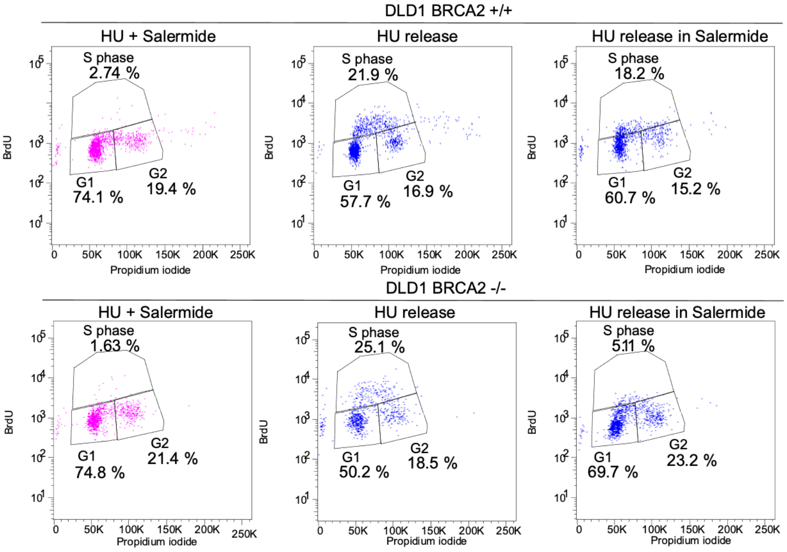

b

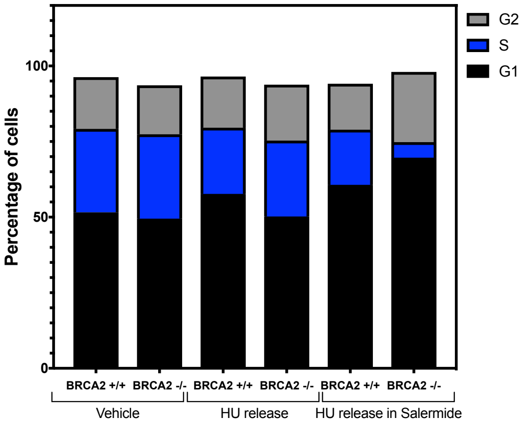

c

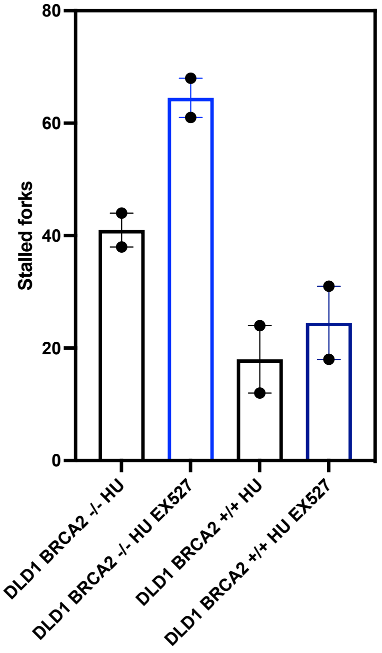

Supplementary Fig. 6. SIRT inhibition causes replication stress in BRCA2 defective cells. a Isogenic DLD1 cells were pre-treated with salermide for 24 hours, cells were then exposed to 2 mM hydroxyurea for two hours and grown without hydroxyurea for 4 hours in media containing 10 µM BrdU. BrdU immunostaining was then performed, alongside propidium iodide, followed by FACS analysis. **b** The percentage of DLD1 *BRCA2*$^{+/+}$ or DLD1 *BRCA2*$^{-/-}$ cells in G1, active S and G2- phases is shown from **a**. Data are representative of two biological replicates. **c** Isogenic DLD1 cells as shown in **Fig. 2f** pre-treated with EX527 or vehicle for 24 hours and exposed to either 2 mM hydroxyurea or DMSO for 2 hours were analysed by DNA fibre assays. IdU-only fibers were quantified. Quantification of stalled forks from two biological replicates are shown. Source data are provided in Supplementary Data 8.

Supplementary Fig. 7

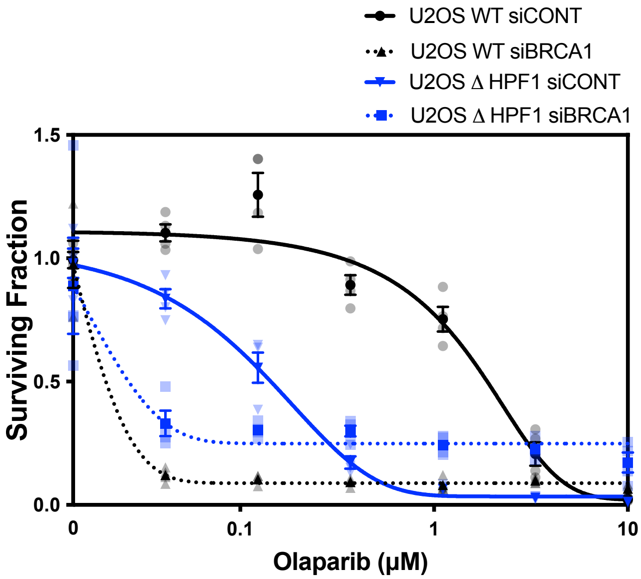

Supplementary Fig. 7 BRCA1 loss sensitizes wild type and HPF1 defective cells to PARP inhibitor. Isogenic U2OS cells were transfected with non-targeting or BRCA1-targeting siRNAs, and were subjected to increasing concentrations of olaparib for six days. Cell viability was assessed using CellTiter-Glo, and surviving fractions were calculated, normalised to DMSO, and then analysed using an ANOVA with a Bonferroni correction for multiple comparisons. Error bars, SEM from four experiments. Data are reflective of two biological replicates**.** Source data are provided in Supplementary Data 8.

Supplementary Fig. 8

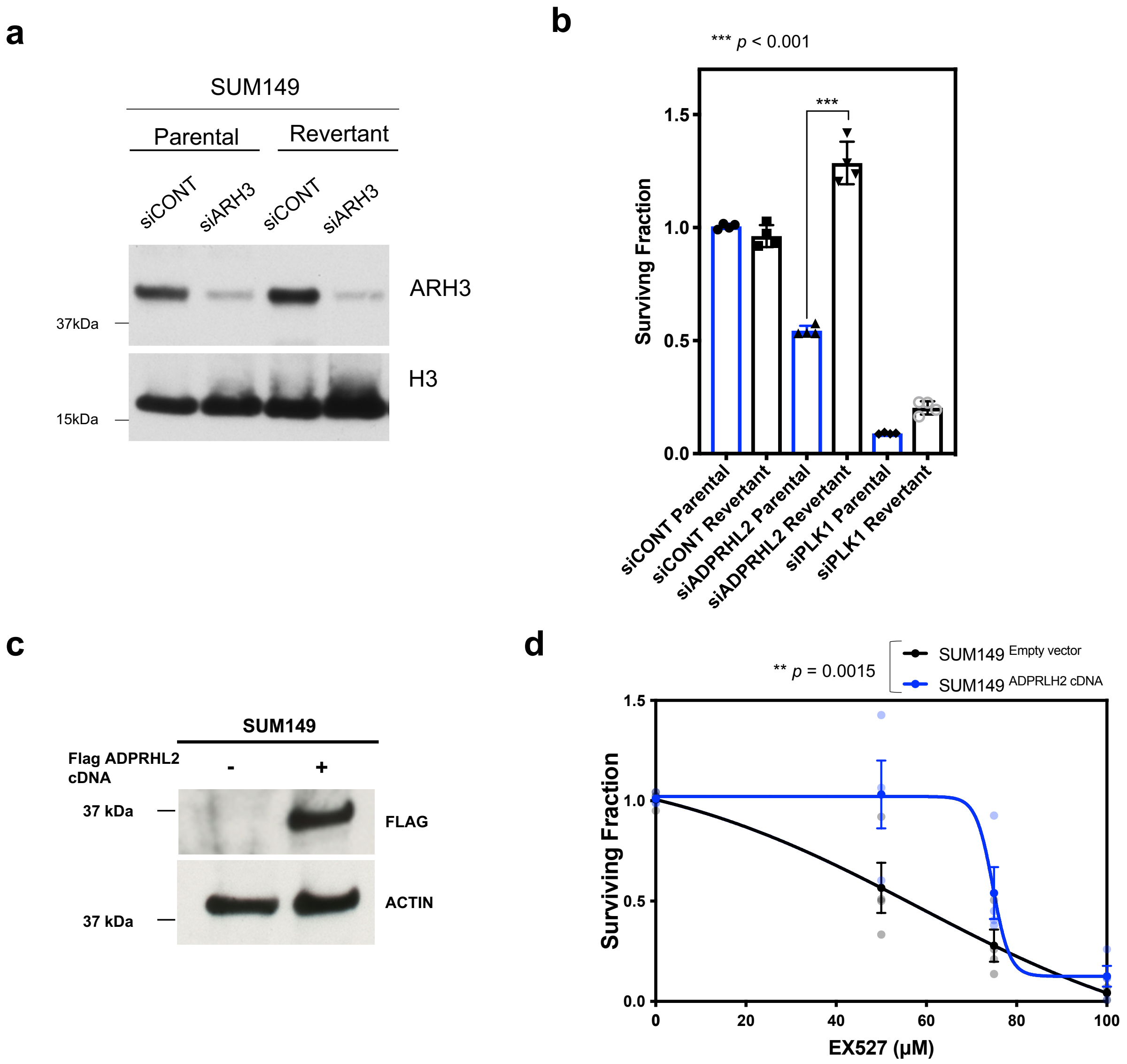

Supplementary Fig. 8. *ADPRHL2* overexpression rescue the SIRT/BRCA SL. a Isogenic SUM149 cell lysates transfected with non-targeting or *ADPRHL2*-targeting siRNAs were analysed by western blotting using anti-ARH3 and anti-H3 antibodies. **b** Isogenic SUM149 cell were transfected with siRNAs targeting *ADPRHL2* or a non targeting control siCONT siRNA. Seven days post transfection, cell viability was assessed using CellTiter-Glo, and values normalised to viability following control siRNA transfection. Data presented as surviving fraction, relative to siRNA control, and analysed using a Student's t-test. Error bars, SEM from four independent experiments. **c** SUM149 cells transfected with a control vector or a vector encoding for *ADPRHL2* cDNA. Cell lysates were analysed by western blotting using anti-HPF1 and anti-ACTIN antibodies. **d** SUM149 cells transfected with a control vector or a vector encoding for *ADPRHL2* cDNA, and were subjected to increasing concentrations of EX527 for six days. Cell viability was assessed using CellTiter-Glo, and surviving fractions were calculated, normalised to DMSO, and then analysed using an ANOVA with a Bonferroni correction for multiple comparisons. Error bars, SEM from n = 4 independent experiments. Data are reflective of two biological replicates. Source data are provided in Supplementary Data 8.

Supplementary Fig. 9

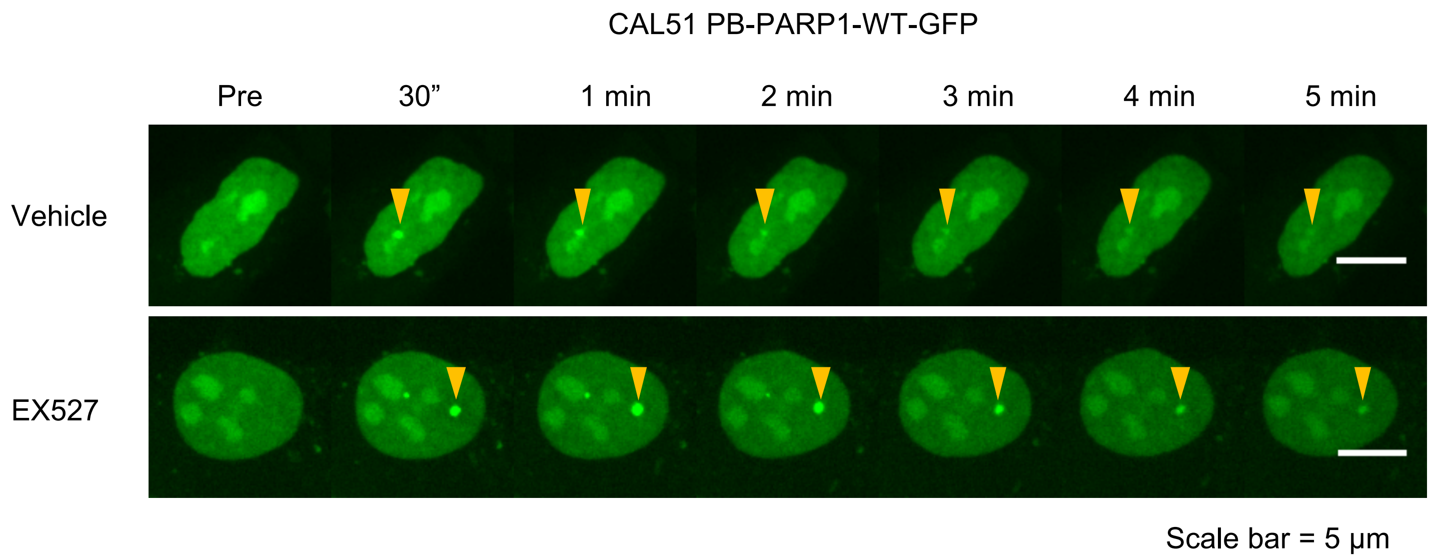

Supplementary Fig. 9. SIRT inhibition leads to increased PARP1 residence on DNA. Representative images of microirradiation experiment as shown in **Fig 5c.** Localisation of PARP1-GFP to damaged DNA was estimated by visualizing GFP signal at the microirradiated spot. The time course of PARP1-GFP signals from CAL51 *PARP1*$^{-/-}$ cells transfected with PARP1-GFP is shown for vehicle (left) and EX527 (right) exposed cells. Source data are provided in Supplementary Data 8.

Supplementary Fig. 10

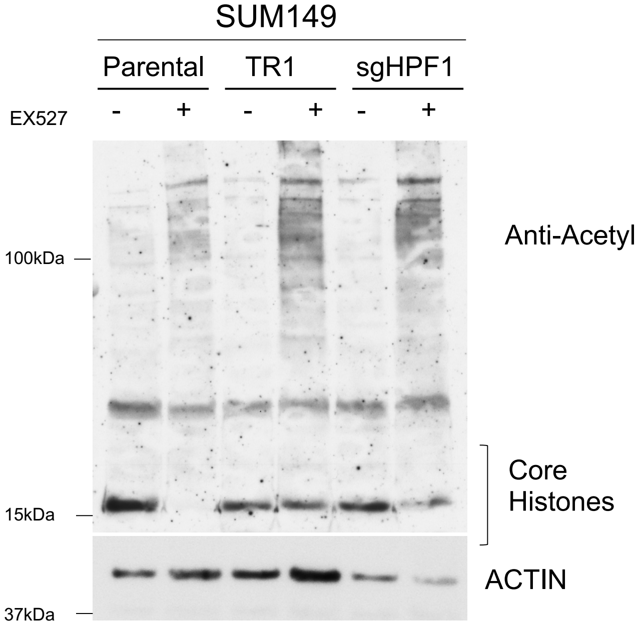

Supplementary Fig. 10. SIRT inhibition leads to PARP1- and HPF1-dependent decreased core histone acetylation. Parental, *PARP1* mutant SUM149 (TR1) or HPF1 defective cells were exposed to 75 μM EX527 for 48 hours and subsequently cell lysates were analysed by western blotting using anti-acetyl and anti-ACTIN antibodies.

Supplementary Fig. 11

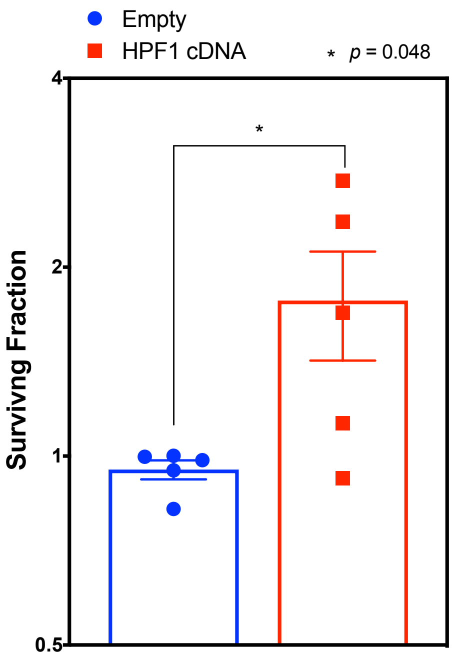

Supplementary Fig. 11. HPF1 over expression promotes cell survival in SUM149 *BRCA1* wild type cells. SUM149 *BRCA1* wild type (revertant) cells were transfected with a control vector or a vector encoding for HPF1 cDNA. Seven days post transfection cell viability was assessed using CellTiter-Glo, and surviving fractions were calculated, normalised to control empty vector. Data was analysed using a Student's t-test. Error bars, SEM from five independent experiments. Source data are provided in Supplementary Data 8.

Supplementary Fig. 12

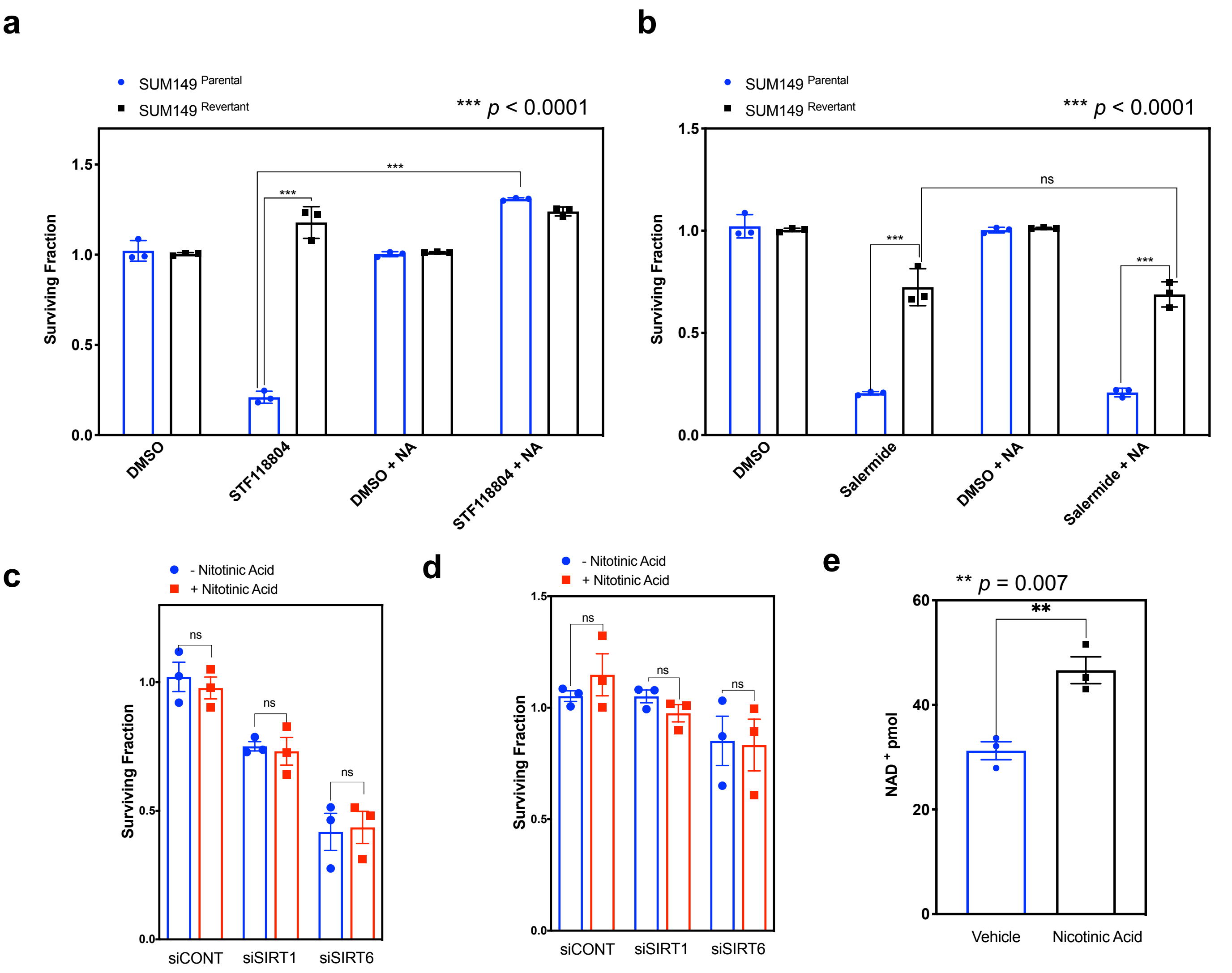

Supplementary Fig. 12. NAD$^+$ supplementation *via* nicotinic acid exposure does not rescue the SIRT inhibitor sensitivity of *BRCA*-gene defective cells. a Isogenic SUM149 cells were exposed to 10 µM STF118804 (NAMPT inhibitor), DMSO control or a combination of 10 µM STF118804 and 10 µM Nicotinic Acid (NA), DMSO and 10 µM Nicotinic Acid (NA) control, and cultured for six subsequent days. Data presented as surviving fraction, relative to DMSO or DMSO+NA control, and analysed using a Student's t-test (n = 3). **b** Isogenic SUM149 cells were exposed to 10 µM salermide, DMSO control or a combination of 10 µM salermide and 10 µM Nicotinic Acid (NA), DMSO and 10 µM Nicotinic Acid (NA) control, and cultured for six subsequent days. Data presented as surviving fraction, relative to DMSO or DMSO NA control, and analysed using a Student's t-test (n = 3). **c** SUM149 parental cells were transfected with siRNAs targeting SIRTs 1, 6 or a non-targeting control siRNA (siCONT) and exposed to either vehicle or 10 µM Nicotinic Acid (NA). Seven days post transfection, cell viability was assessed using CellTiter-Glo, and values normalised to viability following control siRNA transfection. Data presented as surviving fraction, relative to siRNA control, and analysed using a Student's t-test. Error bars, SEM from three independent experiments. **d** SUM149 revertant cells transfected as in **c**. Seven days post transfection, cell viability was assessed using CellTiter-Glo, and values normalised to viability following control siRNA transfection. Data presented as surviving fraction, relative to siRNA control, and analysed using a Student's t-test. Error bars, SEM from three independent experiments. **e** SUM149 Parental cells were exposed to vehicle (DMSO) or 10 µM Nicotinic Acid (NA) for 48 h after which NAD$^+$ levels were estimated using an NAD/NADH Assay Kit (Abcam). Data analysed using a Student's t-test. Error bars, SEM from three independent experiments. Source data are provided in Supplementary Data 8.

Supplementary Fig. 13

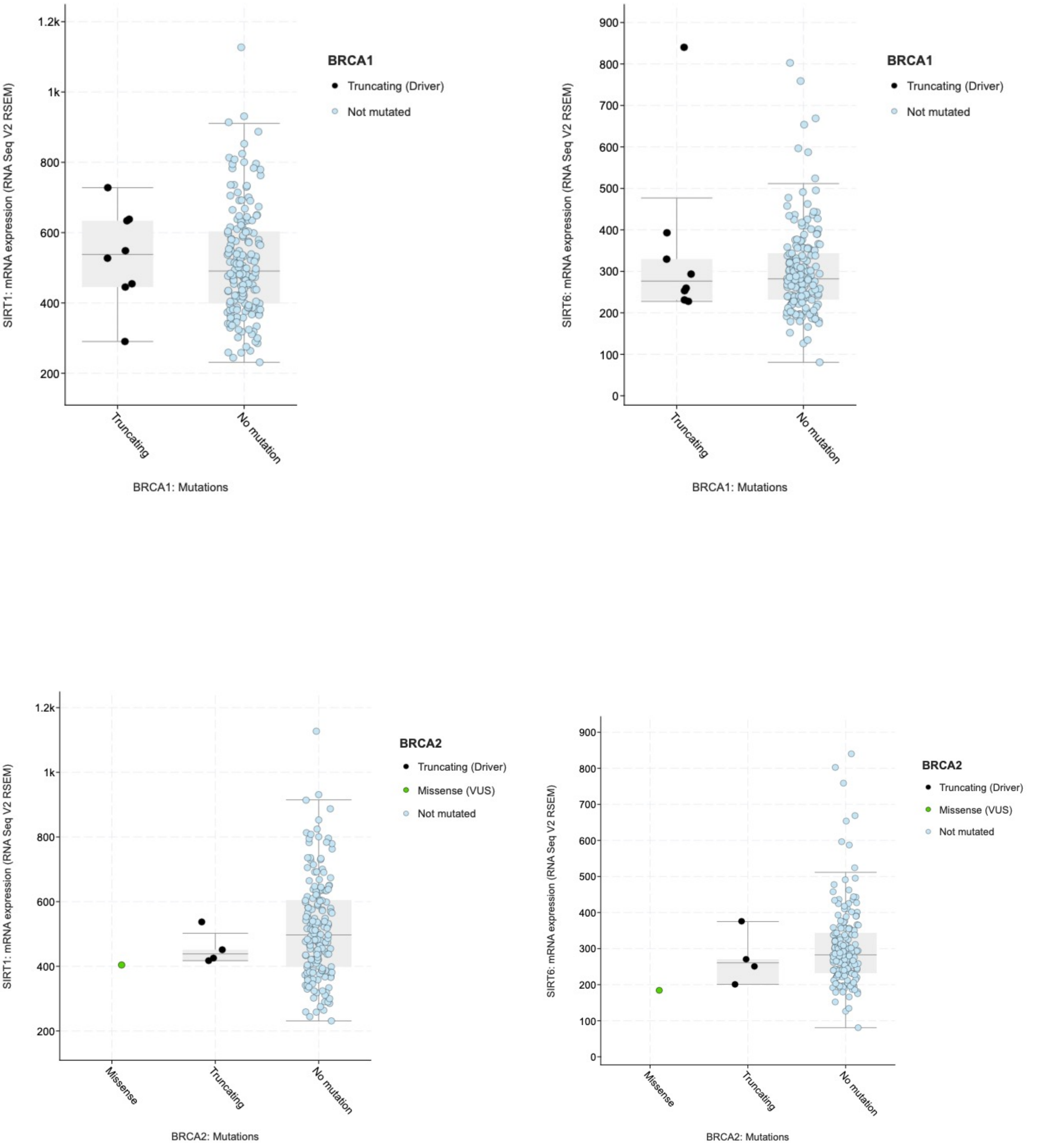

Supplementary Fig. 13. Expression of *SIRT1* and *SIRT6* in *BRCA* mutant ovarian cancers. Ovarian Serous Cystadenocarcinoma (TCGA, Firehouse Legacy). Using genomic sequencing and mRNA expression data from microarray studies of 182 patient tumours (08/04/2021), patients were dichotomised based on *BRCA1* or *BRCA2* mutation status, and *SIRT1* or *SIRT6* expression plotted. No significant alterations in *SIRT1* or *SIRT6* expression were observed in *BRCA1* (p = 0.7333; p = 0.2049) or *BRCA2* (p = 0.3160; p = 0.3444) mutant cases. Source data are provided in Supplementary Data 8.

Supplementary Fig. 14 Uncropped western blots

Fig. 1b

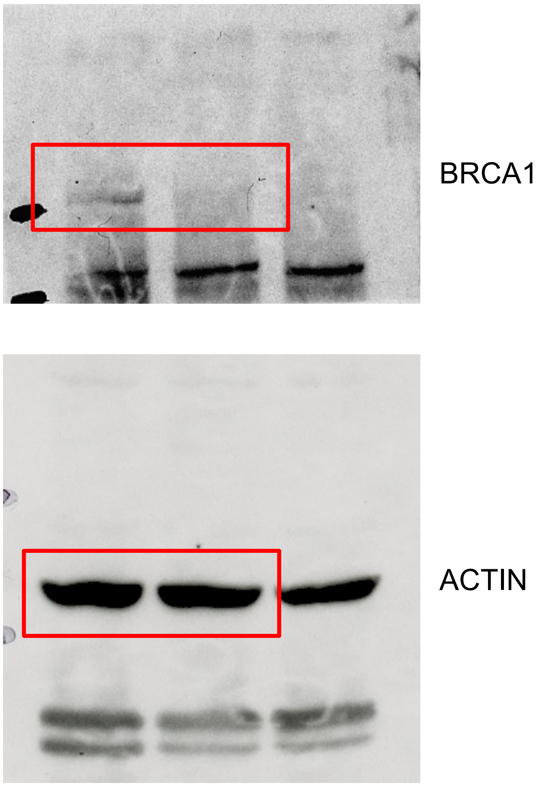

Fig. 1d

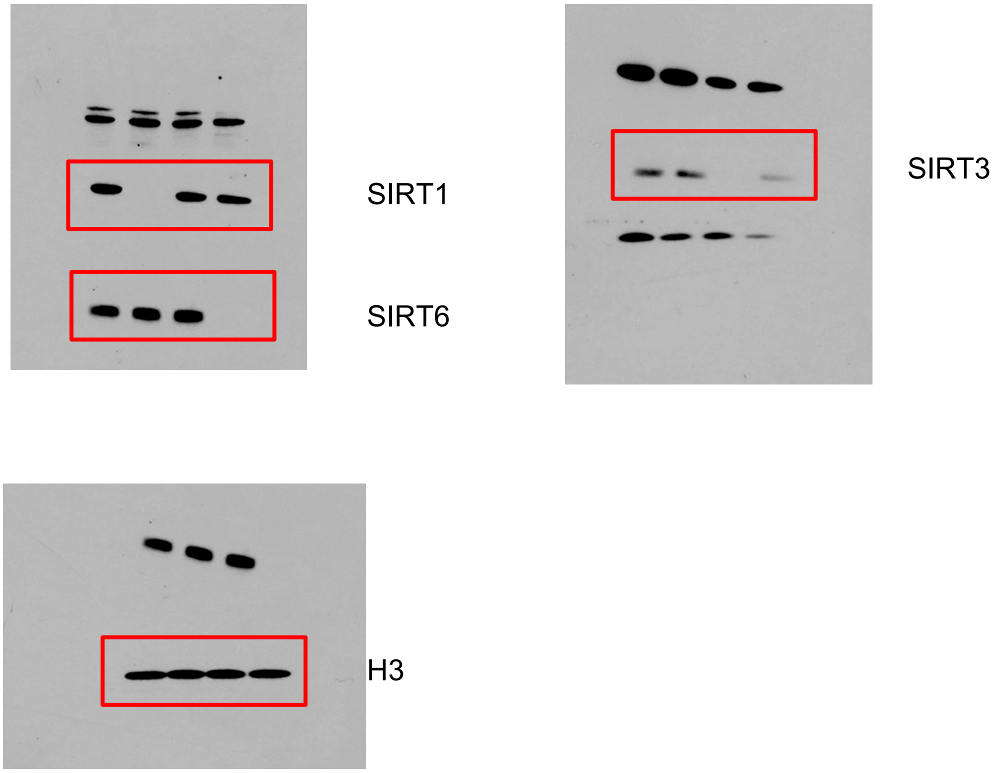

Supplementary Fig. 14 Uncropped western blots continued

Fig. 1h

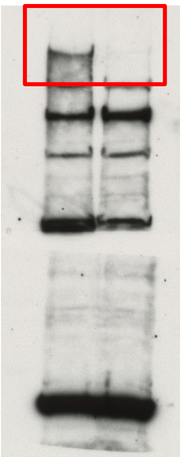

BRCA2

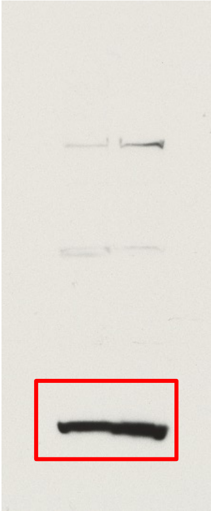

ACTIN

Fig. 1p

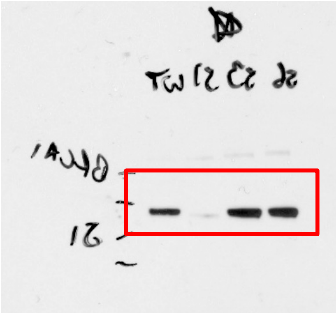

SIRT1

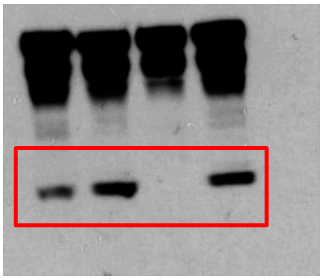

SIRT3

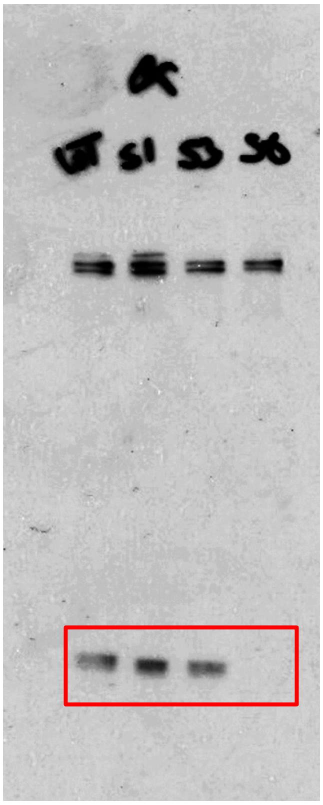

SIRT6

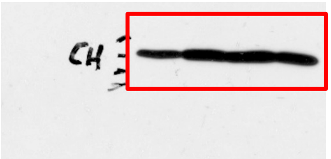

H3

Supplementary Fig. 14 Uncropped western blots continued

Fig. 2a

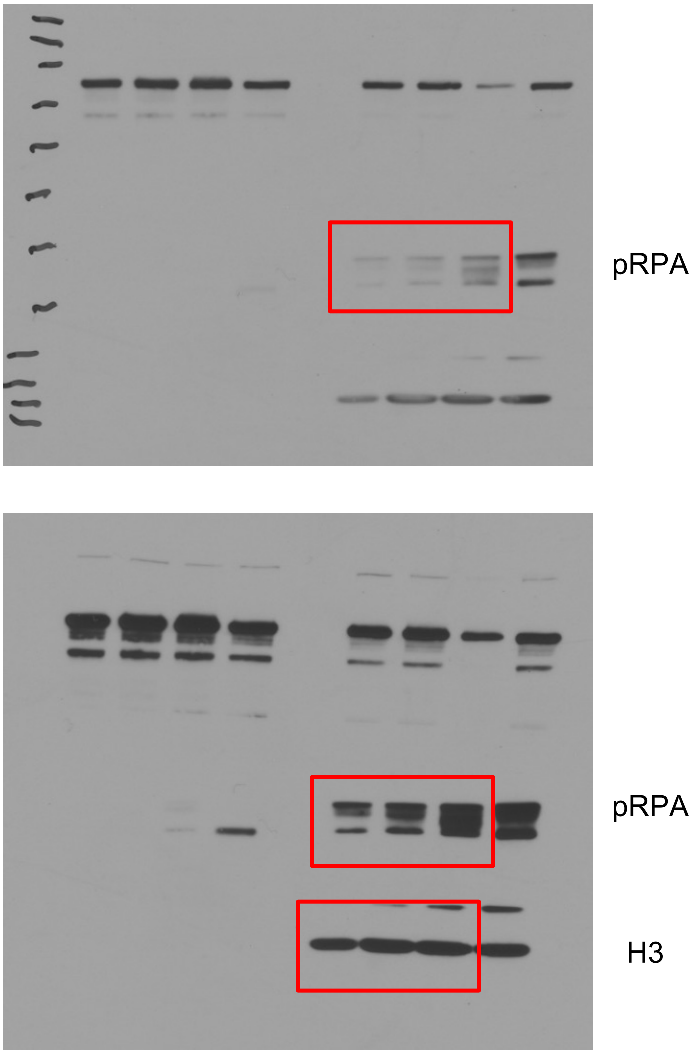

Supplementary Fig. 14 Uncropped western blots continued

Fig. 3d

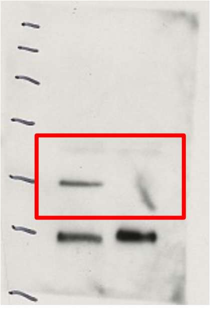

HPF1

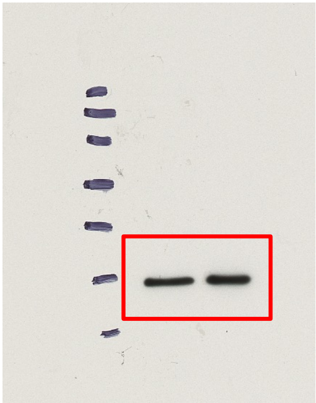

ACTIN

Fig. 3e

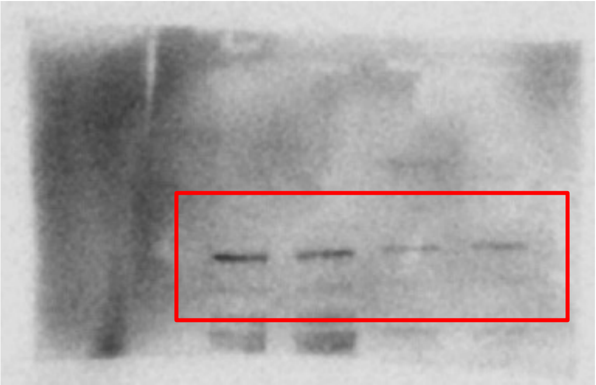

BRCA1

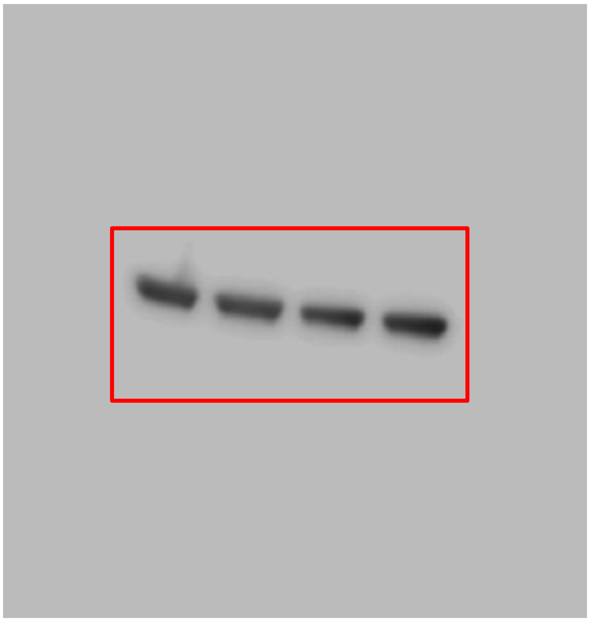

ACTIN

Fig. 4a

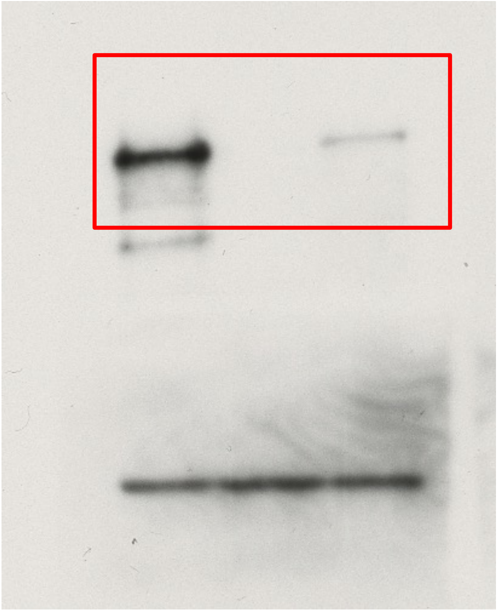

PARP1

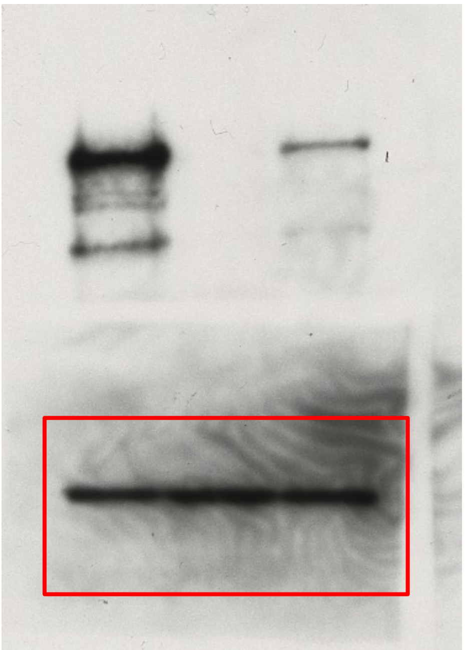

ACTIN

Supplementary Fig. 14 Uncropped western blots continued

Fig. 5a

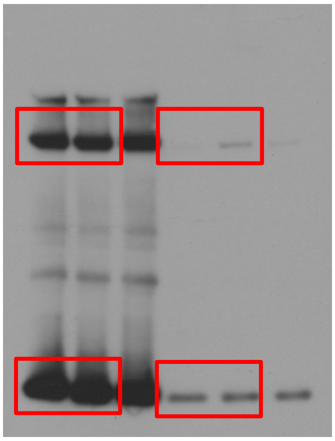

PARP1

PCNA

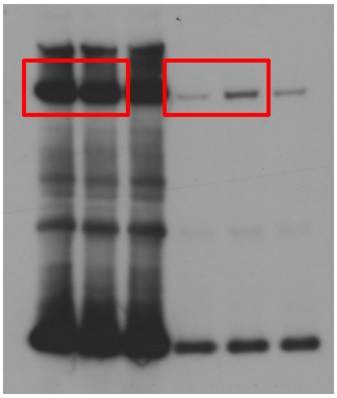

PARP1 high exposure

Fig. 5b

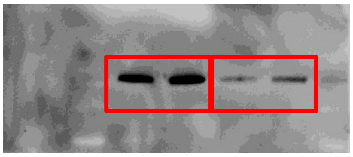

PARP1

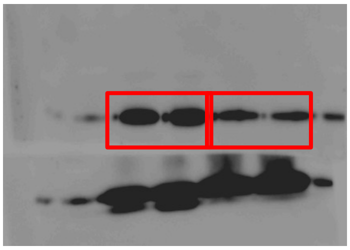

PCNA

Supplementary Fig. 14 Uncropped western blots continued

Fig. 5d

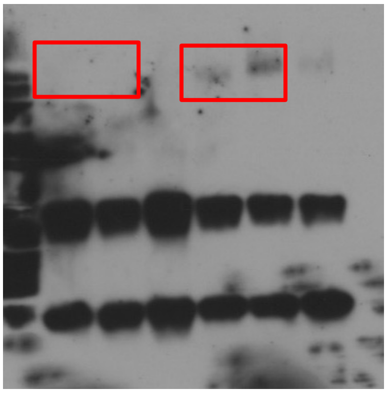

Acetyl

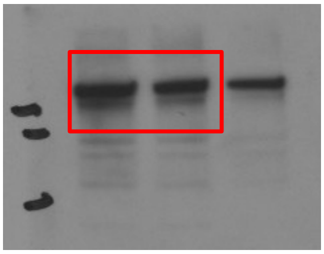

PARP1

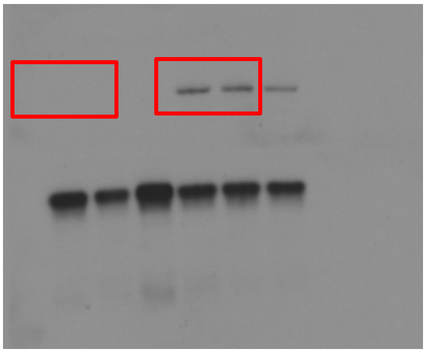

PARP1

First two lanes of western blot below are the same as shown in Fig. 5e

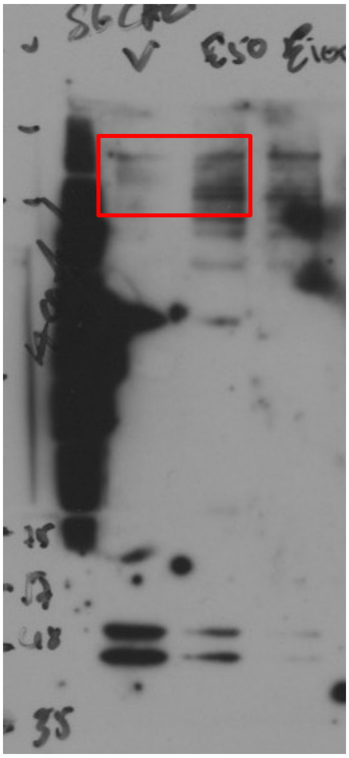

Acetyl

Supplementary Fig. 14 Uncropped western blots continued

Fig. 5e

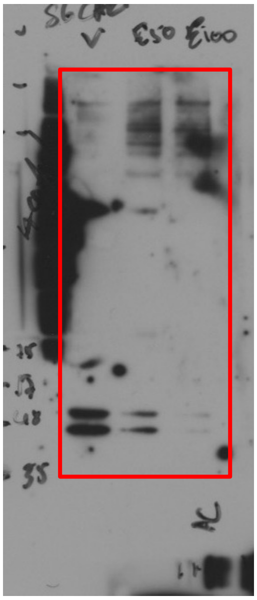

Acetyl

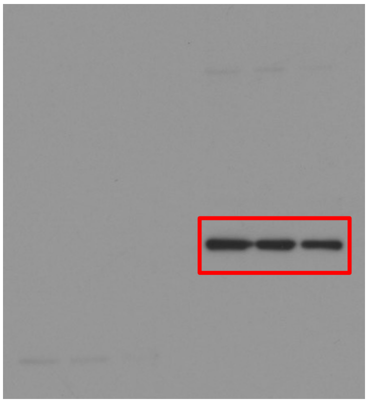

ACTIN

Fig. 5f

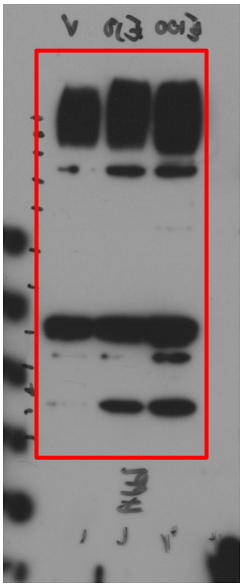

PAR high exposure

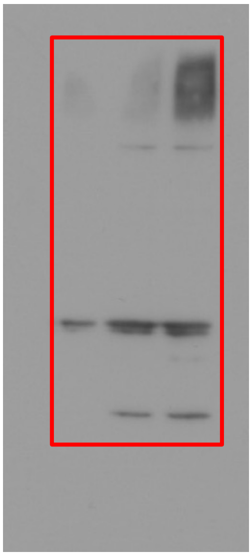

PAR low exposure

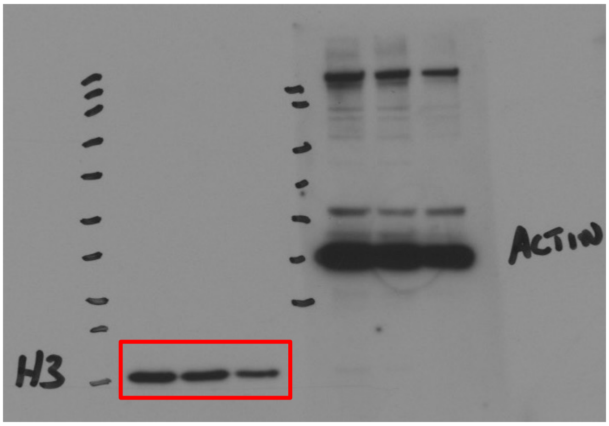

H3

Supplementary Fig. 14 Uncropped western blots continued

Fig. 5g Western blots used for densitometry quantification

Biological repeat 1 | Biological repeat 2 | Biological repeat 3

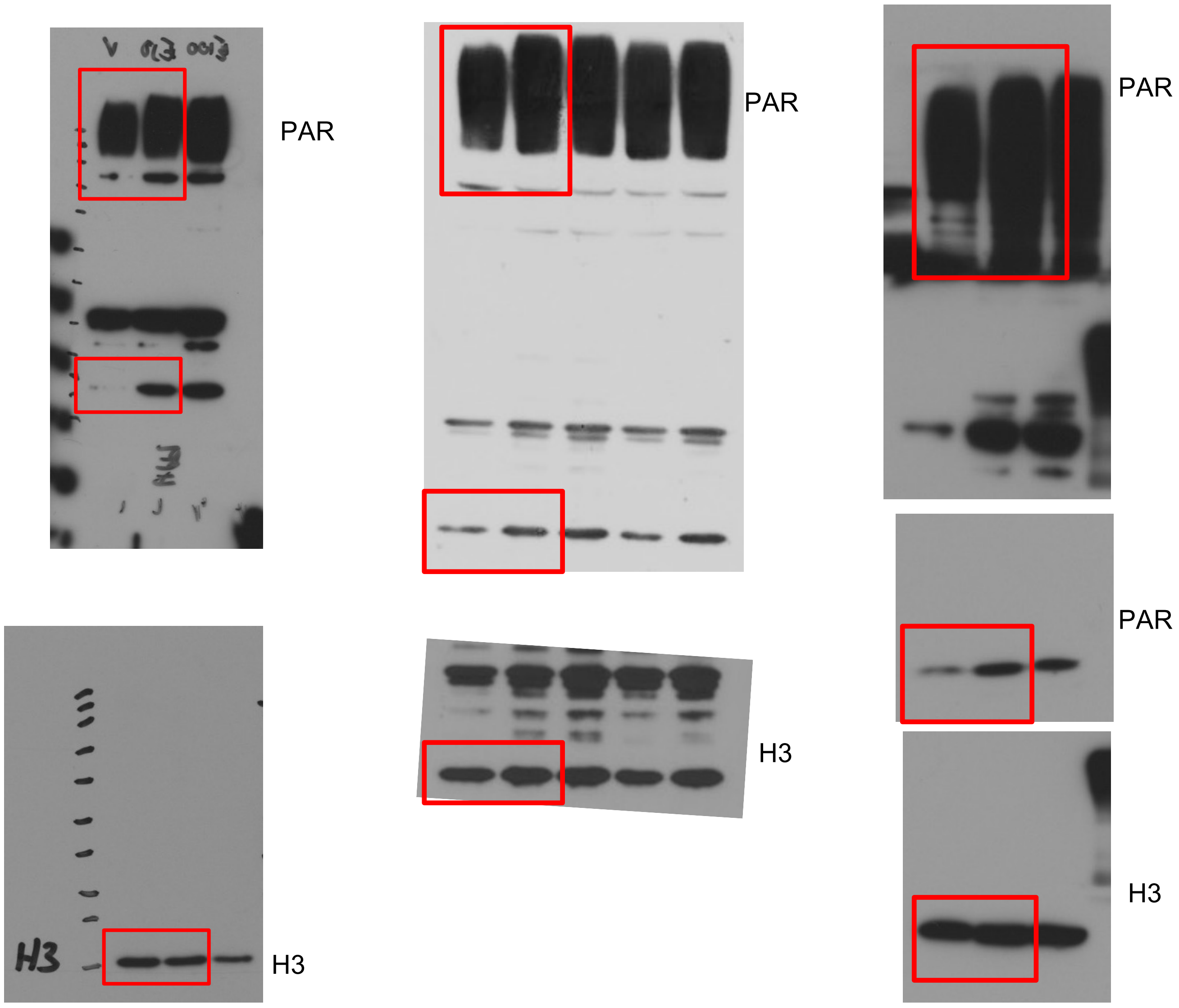

Supplementary Fig. 14 Uncropped western blots continued

Fig. 5h

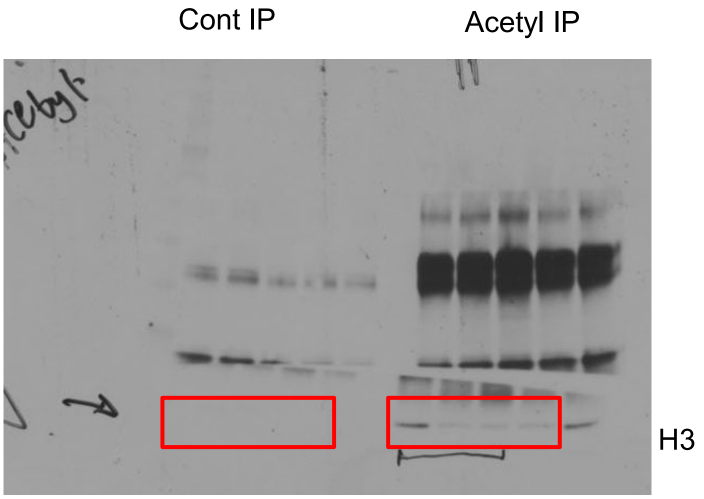

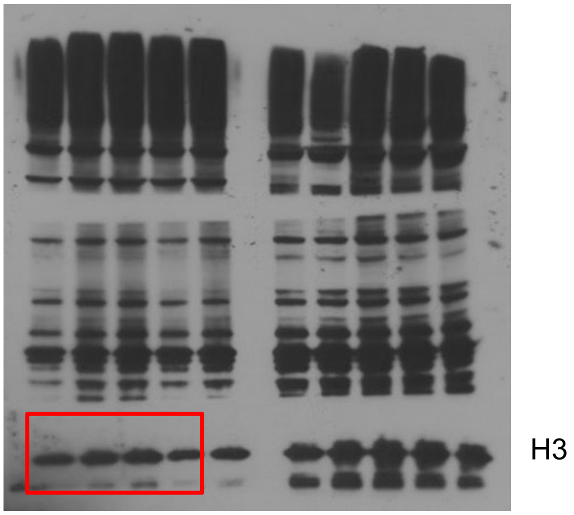

Fig. 5i

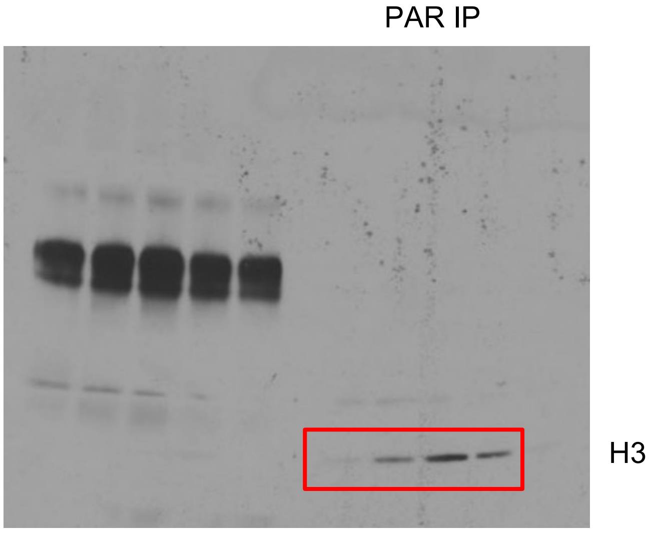

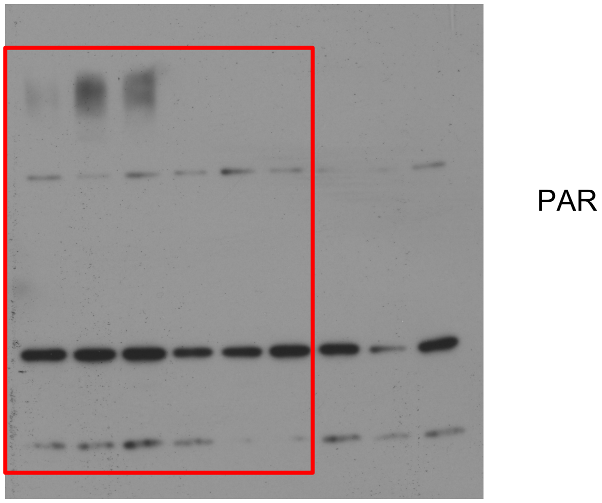

Fig. 5j Western blots used for densitometry quantification

Biological repeat 1

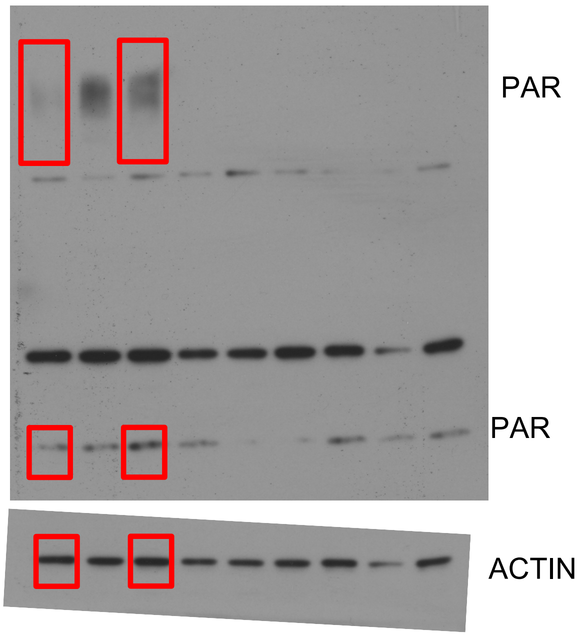

Biological repeat 2

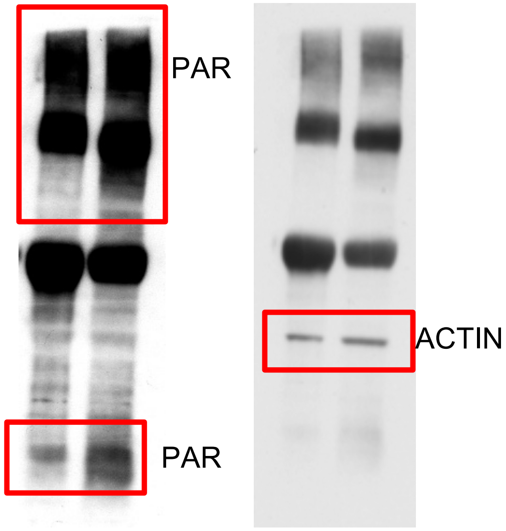

Biological repeat 3

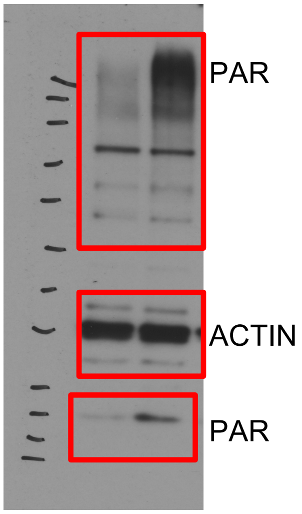

Supplementary Fig. 14 Uncropped western blots continued

Fig. 5k

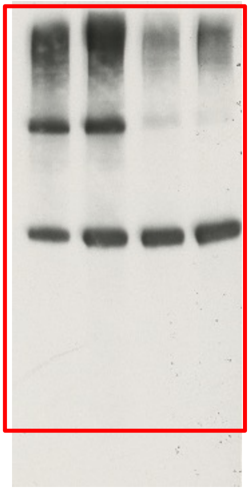

PAR low exposure

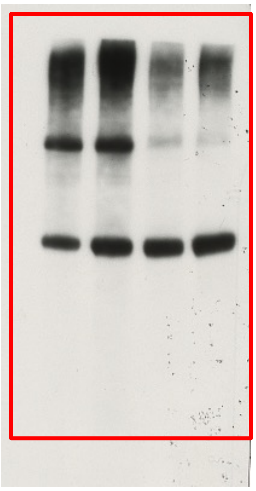

PAR high exposure

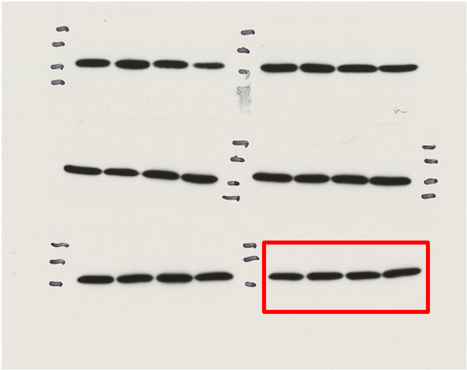

H3

Fig. 5l

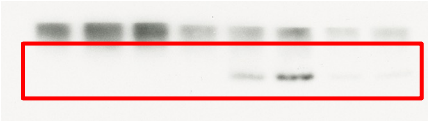

H3 low exposure

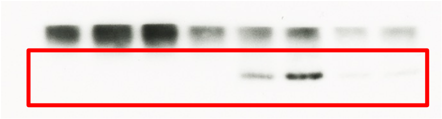

H3 high exposure

Supplementary Fig. 14 Uncropped western blots continued

Fig. 6a

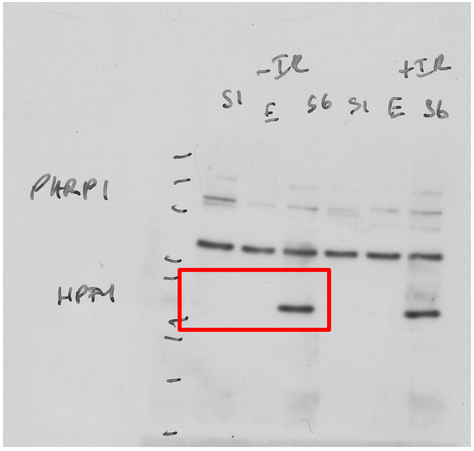

HPF1

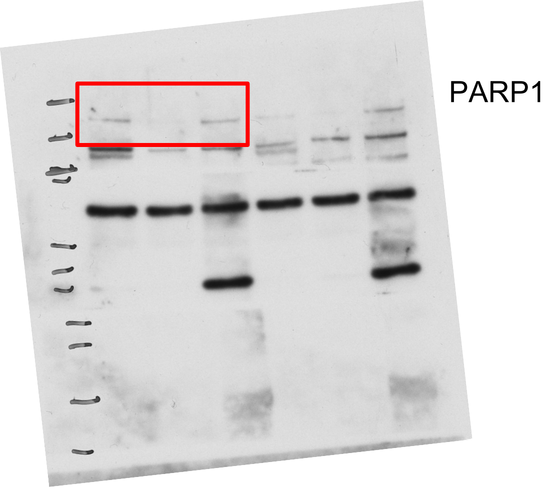

PARP1

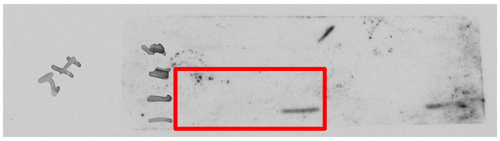

H3

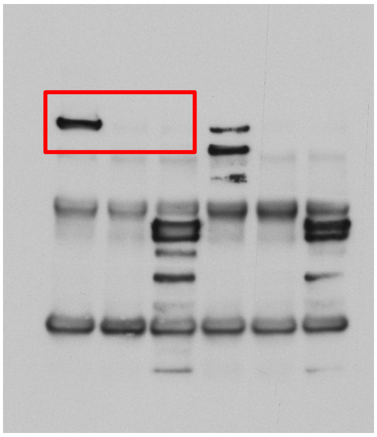

SIRT1

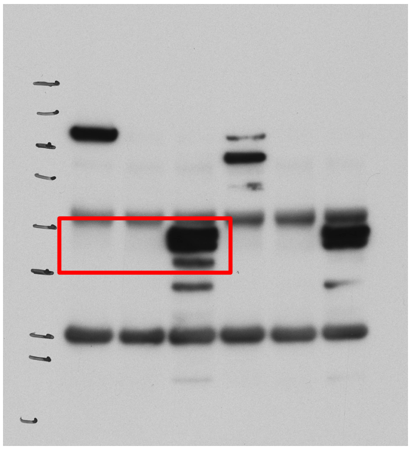

SIRT6

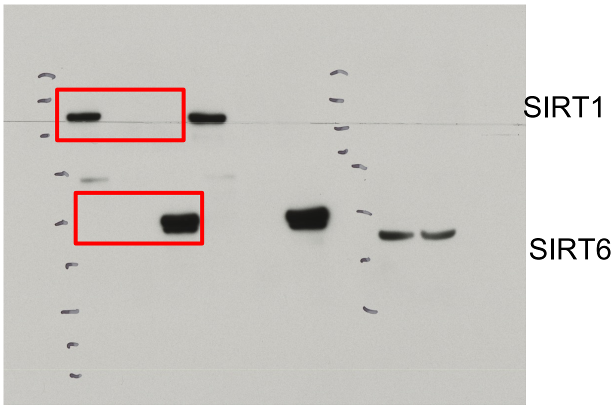

SIRT1

SIRT6

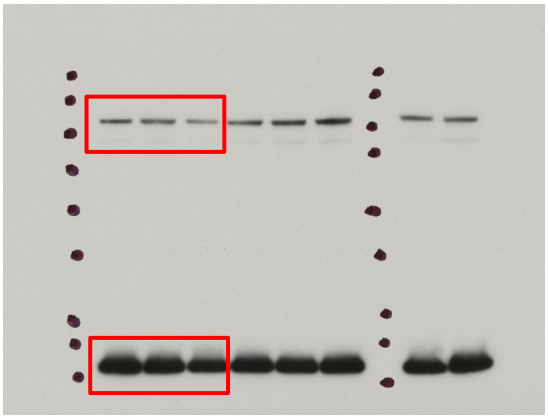

PARP1

H3

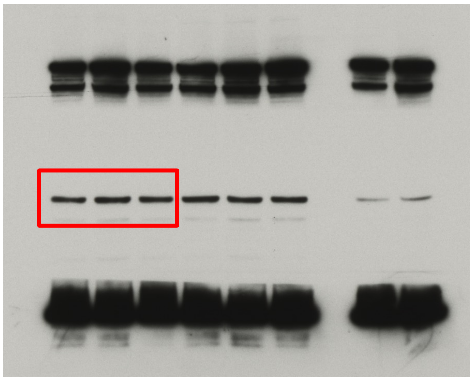

HPF1

Supplementary Fig. 14 Uncropped western blots continued

Fig. 6b

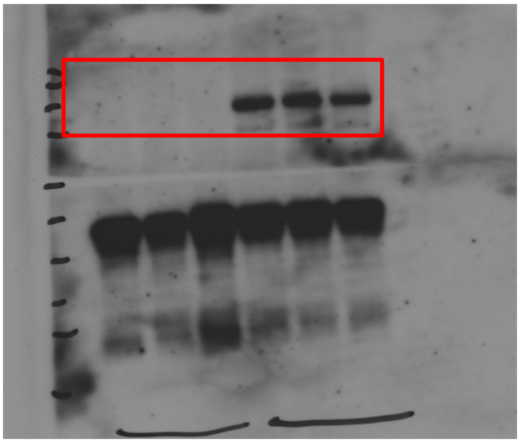

PARP1

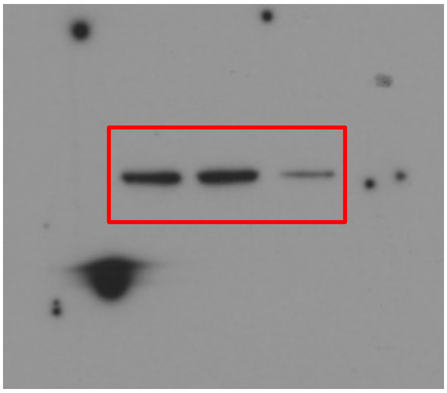

PARP1

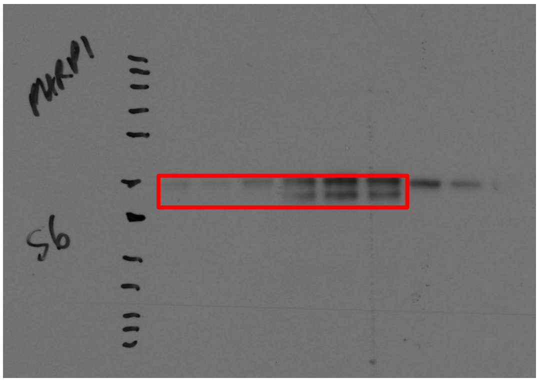

SIRT6

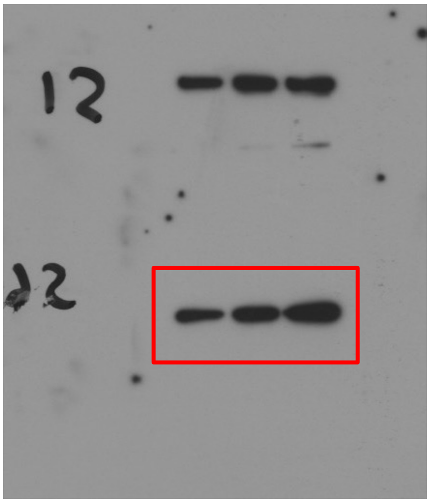

SIRT6

Fig. 6c Western blots used for densitometry quantification in Fig. 6d

Biological repeat 1

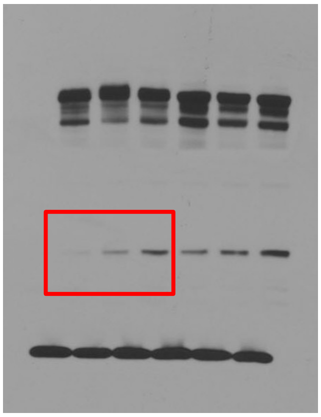

HPF1 low exposure

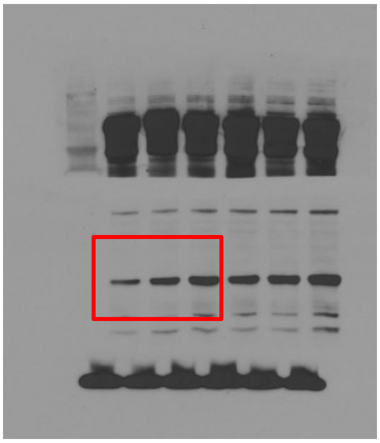

HPF1 high exposure

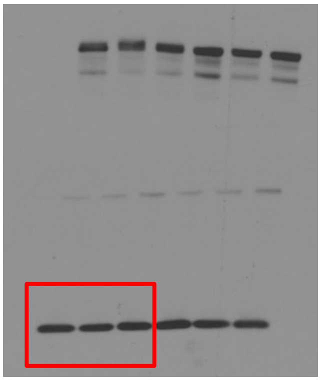

H3

Supplementary Fig. 14 Uncropped western blots continued

Fig. 6d Western blots used for densitometry quantification

Biological repeat 2

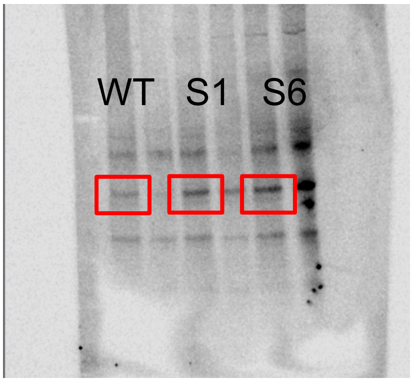

HPF1

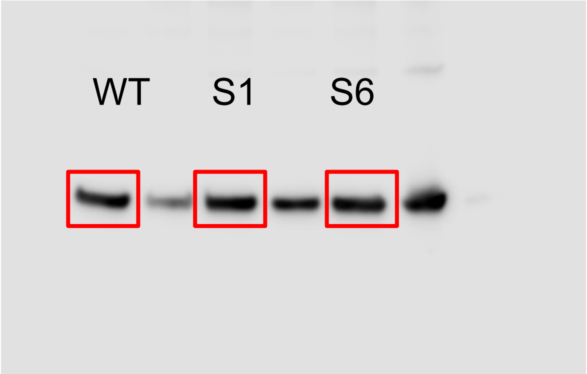

H3

Biological repeat 3

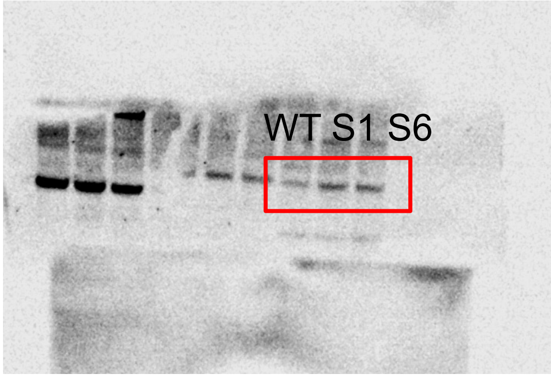

HPF1

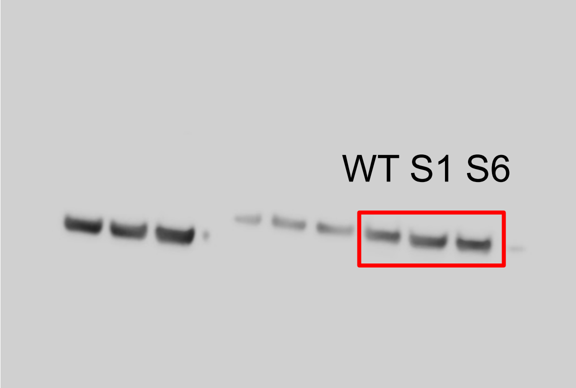

H3

Biological repeat 4

Biological repeat 5

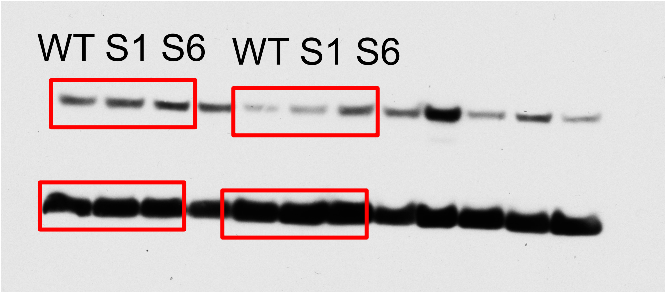

HPF1

H3

Supplementary Fig. 14 Uncropped western blots continued

Fig. 6e

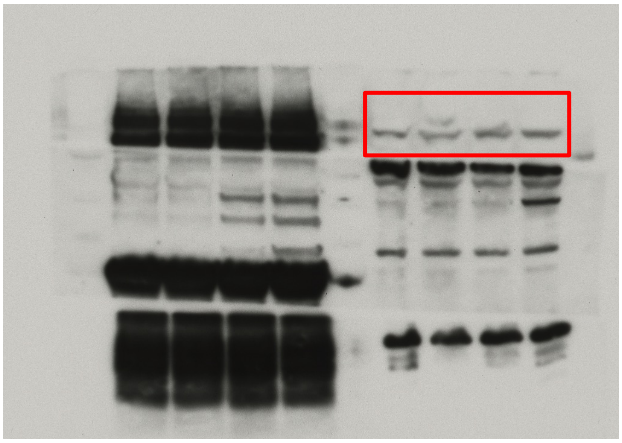

PARP1

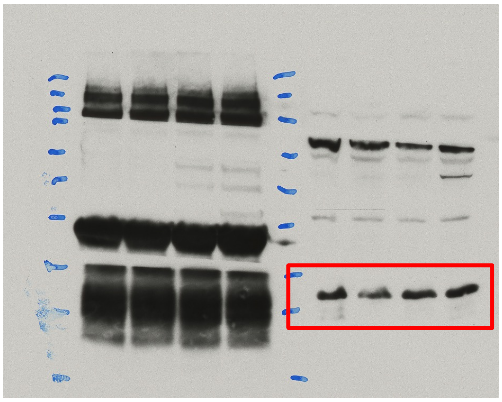

H3

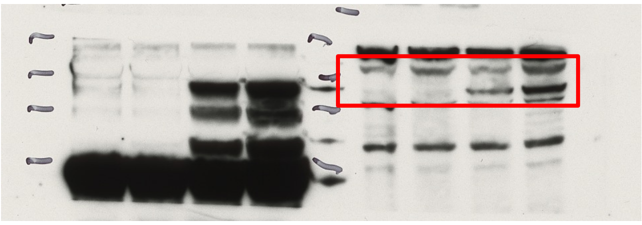

FLAG high exposure

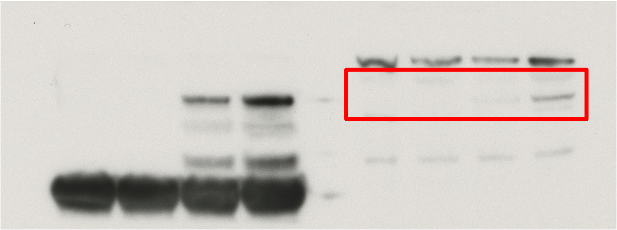

FLAG low exposure

Supplementary Fig. 14 Uncropped western blots continued

Fig. 6f Western blots used for densitometry quantification

Biological repeat 1

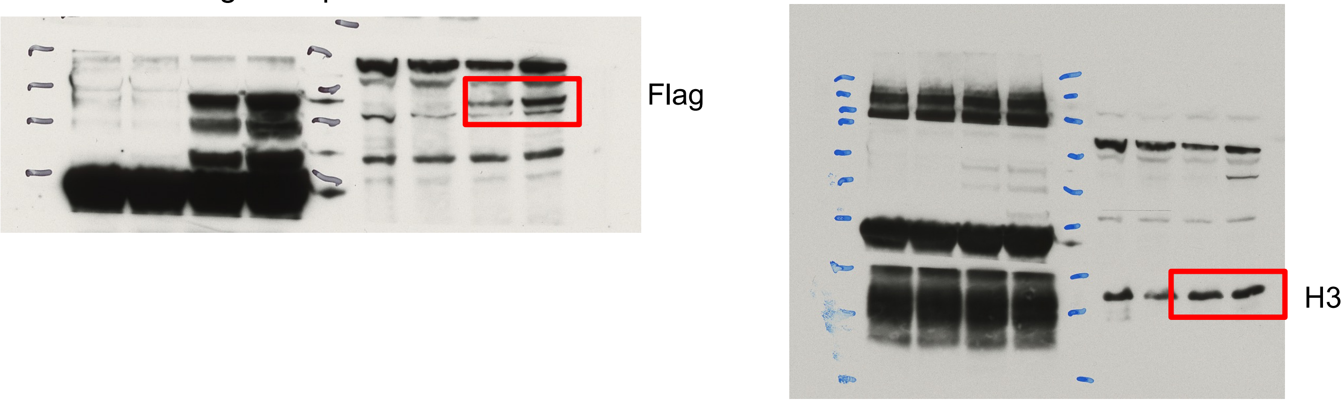

Biological repeat 2

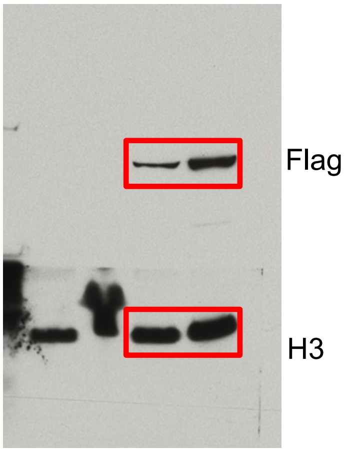

Biological repeat 3

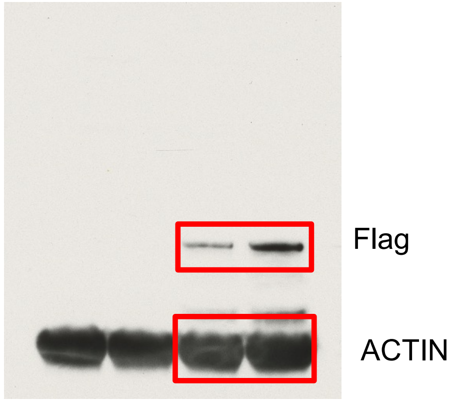

Supplementary Fig. 14 Uncropped western blots continued

Fig. 6g and Fig. 6h

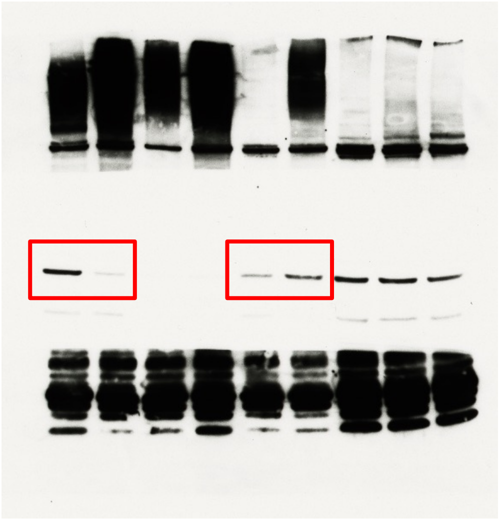

HPF1 high exposure

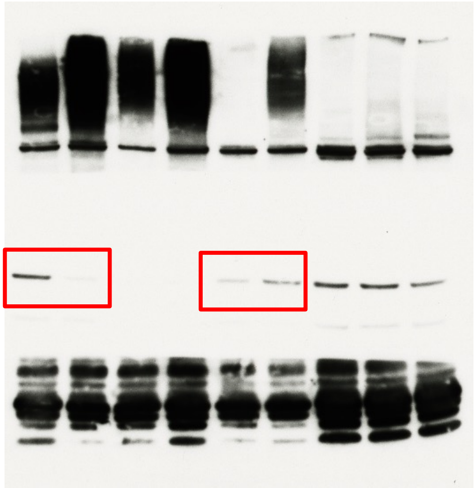

HPF1 low exposure

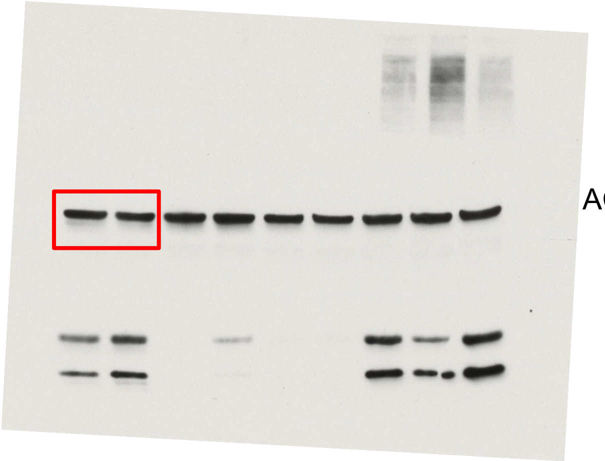

ACTIN

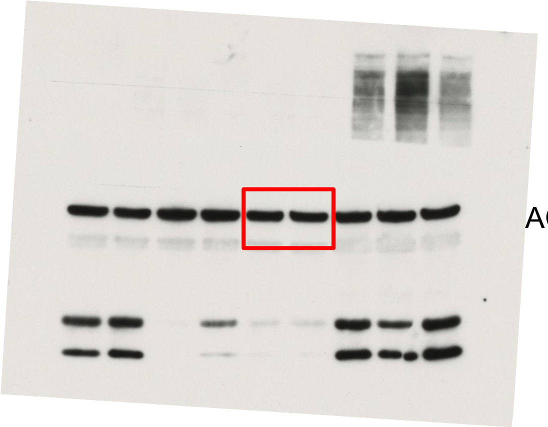

ACTIN

Supplementary Fig. 14 Uncropped western blots continued

Fig. 6h Western blots used for densitometry quantification

Biological repeat 1

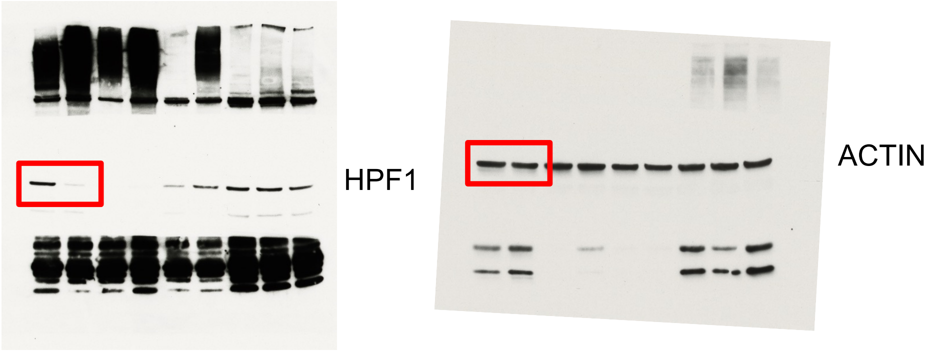

Biological repeat 2

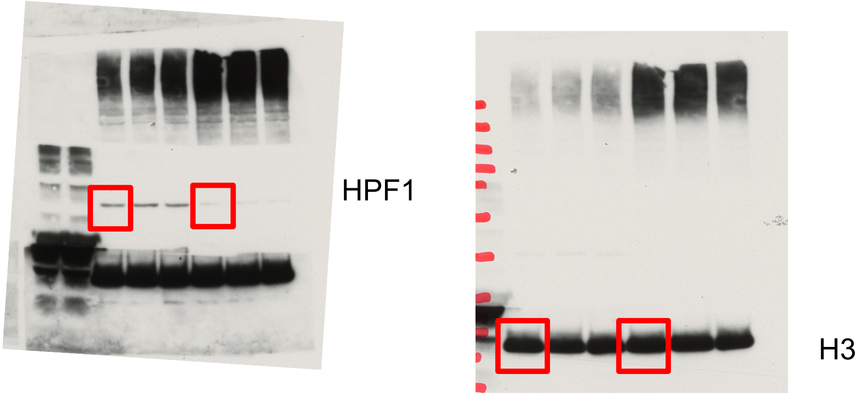

Biological repeat 3

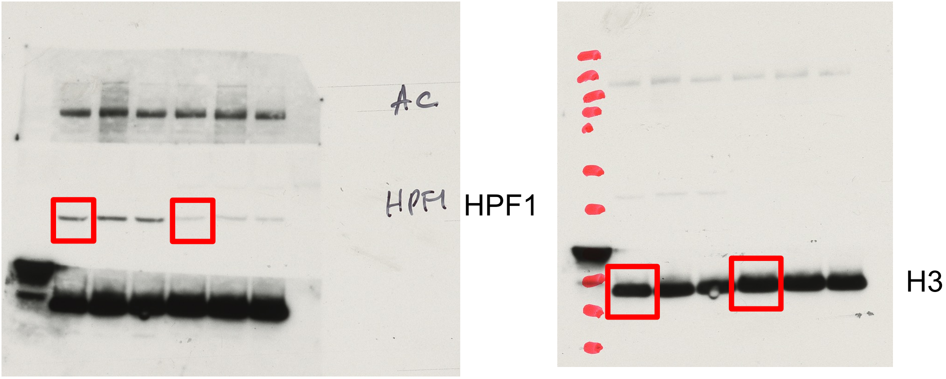

Supplementary Fig. 14 Uncropped western blots continued

Fig. 6i and Fig. 6j Western blots used for densitometry quantification

Biological repeat 1

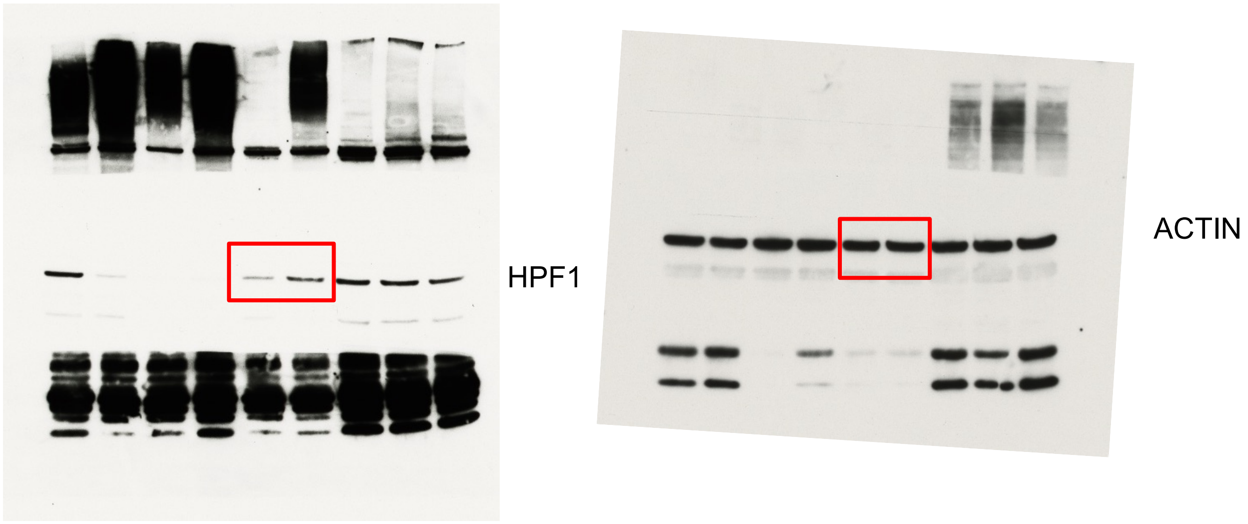

Biological repeat 2

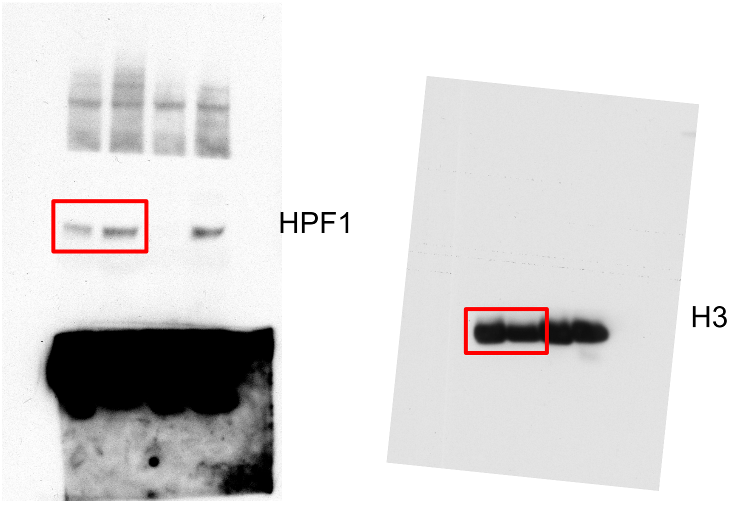

Biological repeat 3

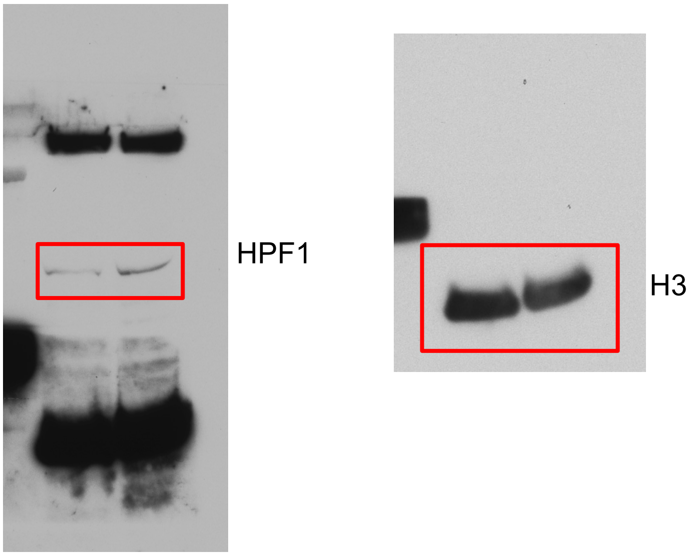

Supplementary Fig. 14 Uncropped western blots continued

Supplementary Fig. 4c

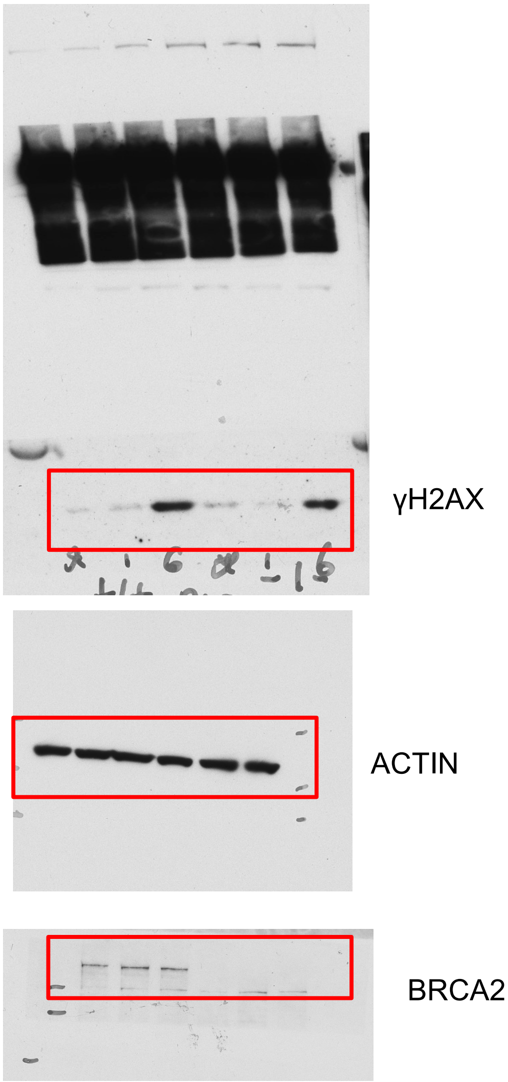

Supplementary Fig. 14 Uncropped western blots continued

Supplementary Fig. 4d

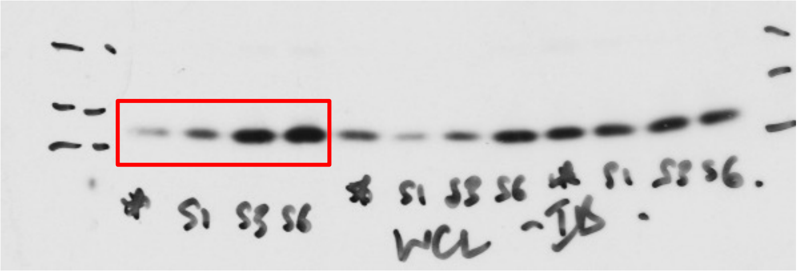

γH2AX

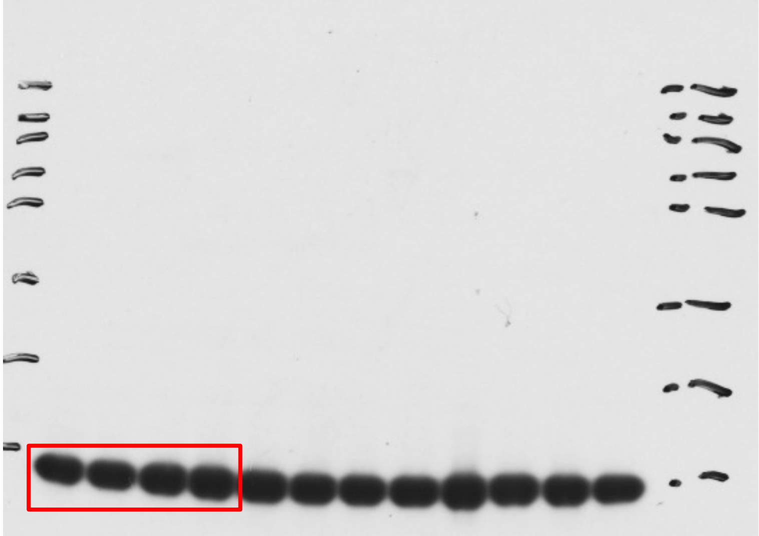

PCNA

Supplementary Fig. 14 Uncropped western blots continued

Supplementary Fig. 8a

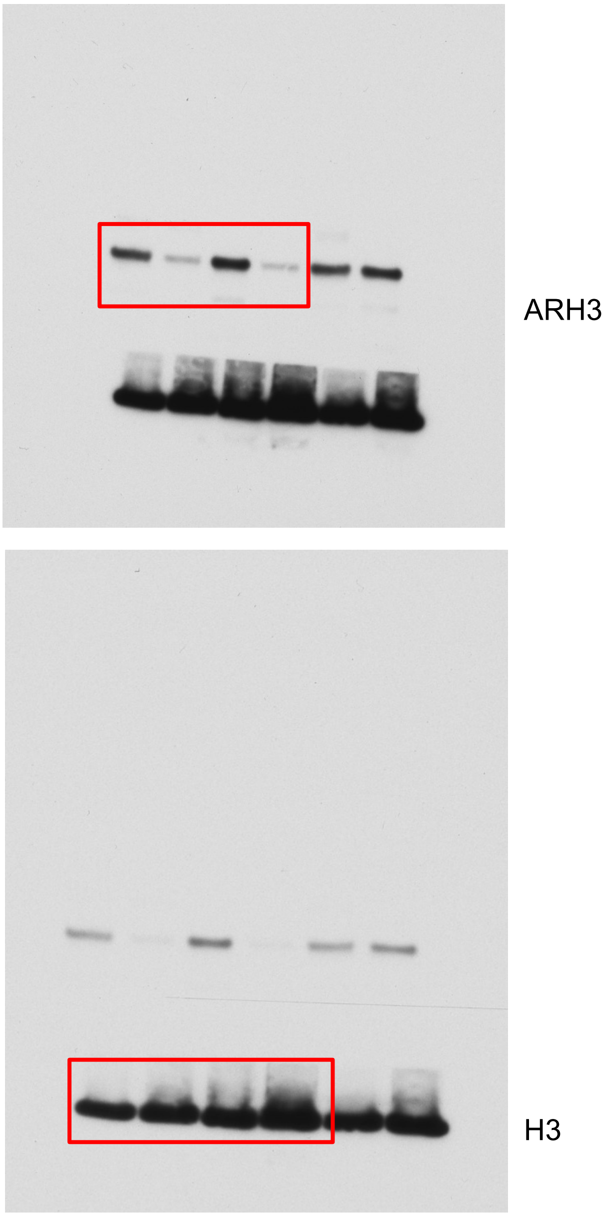

Supplementary Fig. 8c

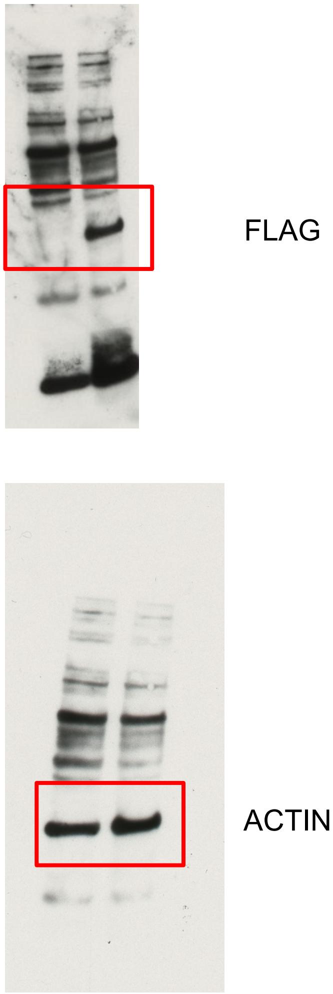

Supplementary Fig. 14 Uncropped western blots continued

Supplementary Fig. 10

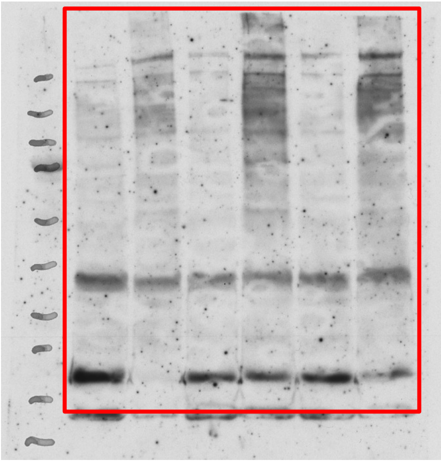

Pan-Acetyl

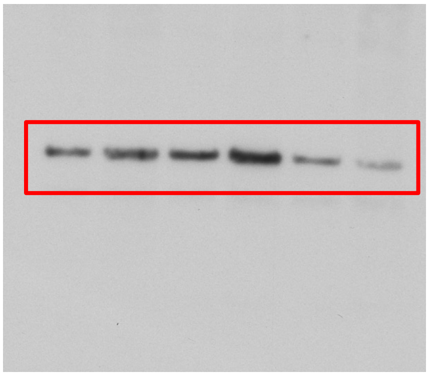

ACTIN
