# Supplementary material for: Sirtuin inhibition is synthetic lethal with BRCA1 or BRCA2 deficiency
Source: Commun Biol. 2021 Nov 8;4:1270. doi: 10.1038/s42003-021-02770-2 (PMC8575930; doi:10.1038/s42003-021-02770-2)
Supplement: Supplementary file 2 — Description of Additional Supplementary Files [file 42003_2021_2770_MOESM2_ESM.pdf]

Description of Additional Supplementary Files

File name: Supplementary Data 1.
Description: Synthetic lethal screen in SUM149 cells.

File name: Supplementary Data 2.
Description: Summary of siRNA causing SIRTi sensitivity in CAL51 cells.

File name: Supplementary Data 3.
Description: GO pathway enrichment analysis of CAL51 screen.

File name: Supplementary Data 4.
Description: CRISPRa screen for EX527 resistance in SUM149 cells.

File name: Supplementary Data 5.
Description: siRNA library targeting DDR and CGC genes.

File name: Supplementary Data 6.
Description: Source data for all graphs in main Figures.

File name: Supplementary Data 7.
Description: Source data for all graphs in Supplementary Figures.
