# Supplementary material for: Sirtuin inhibition is synthetic lethal with BRCA1 or BRCA2 deficiency
Source: Commun Biol. 2021 Nov 8;4:1270. doi: 10.1038/s42003-021-02770-2 (PMC8575930; doi:10.1038/s42003-021-02770-2)
Supplement: Supplementary file 10 — Reporting Summary [file 42003_2021_2770_MOESM10_ESM.pdf]

Corresponding author(s): Christopher J. Lord
Last updated by author(s): 08/04/2021

Reporting Summary

Nature Research wishes to improve the reproducibility of the work that we publish. This form provides structure for consistency and transparency in reporting. For further information on Nature Research policies, see our Editorial Policies and the Editorial Policy Checklist.

Statistics

For all statistical analyses, confirm that the following items are present in the figure legend, table legend, main text, or Methods section.

n/a	Confirmed	
☐	☒	The exact sample size (*n*) for each experimental group/condition, given as a discrete number and unit of measurement
☐	☒	A statement on whether measurements were taken from distinct samples or whether the same sample was measured repeatedly
☐	☒	The statistical test(s) used AND whether they are one- or two-sided<br>*Only common tests should be described solely by name; describe more complex techniques in the Methods section.*
☒	☐	A description of all covariates tested
☒	☐	A description of any assumptions or corrections, such as tests of normality and adjustment for multiple comparisons
☐	☒	A full description of the statistical parameters including central tendency (e.g. means) or other basic estimates (e.g. regression coefficient) AND variation (e.g. standard deviation) or associated estimates of uncertainty (e.g. confidence intervals)
☐	☒	For null hypothesis testing, the test statistic (e.g. F, t, r) with confidence intervals, effect sizes, degrees of freedom and P value noted<br>*Give P values as exact values whenever suitable.*
☒	☐	For Bayesian analysis, information on the choice of priors and Markov chain Monte Carlo settings
☒	☐	For hierarchical and complex designs, identification of the appropriate level for tests and full reporting of outcomes
☒	☐	Estimates of effect sizes (e.g. Cohen's d, Pearson's r), indicating how they were calculated

Our web collection on statistics for biologists contains articles on many of the points above.

Software and code

Policy information about availability of computer code

Data collection	LSRII<br>CellTitre-Glo luminescence: Perkin Elmer VICTOR X Luminescence Plate Reader<br>Andor Revolution system (Spinning disk confocal)<br>ImageXpress Micro Confocal High-Content Imaging System
Data analysis	Flow Cytometry data analysis: FlowJo version 10<br>Data visualization and statistical analysis: GraphPad Prism Version 9.0<br>Image analysis: ImageJ Fiji version 1.0<br>CRISPR/Cas9 screen data analysis: Drug Effect Z (DE) (see methods in manuscript); MAGeCK (https://sourceforge.net/p/mageck/wiki/Home/)

For manuscripts utilizing custom algorithms or software that are central to the research but not yet described in published literature, software must be made available to editors and reviewers. We strongly encourage code deposition in a community repository (e.g. GitHub). See the Nature Research guidelines for submitting code & software for further information.

Data

Policy information about availability of data

All manuscripts must include a data availability statement. This statement should provide the following information, where applicable:

- Accession codes, unique identifiers, or web links for publicly available datasets
- A list of figures that have associated raw data
- A description of any restrictions on data availability

The results of the siRNA screens targeting NAD+ metabolism enzymes, EX527 CRISPR screen and EX527 chemosensitivity siRNA screen are now provided as source data and supplementary table 1-3 in the online version of the manuscript. Unprocessed images of all immunoblots are presented in Supplementary Fig 14. All other datasets generated during in this study are available from the corresponding authors upon reasonable request.

Field-specific reporting

Please select the one below that is the best fit for your research. If you are not sure, read the appropriate sections before making your selection.

- [x] Life sciences
- [ ] Behavioural & social sciences
- [ ] Ecological, evolutionary & environmental sciences

For a reference copy of the document with all sections, see nature.com/documents/nr-reporting-summary-flat.pdf

Life sciences study design

All studies must disclose on these points even when the disclosure is negative.

Sample size	No statistical methods were used to predetermine sample size. Unless indicated otherwise in the figure legends sample sizes of at least 3 independent experimental replicates were used based on previous experienece and the standard practices of the field.
Data exclusions	No data were excluded from experiments presented in this manuscript.
Replication	All experiments were performed in at least biological triplicate with similar results. Where only two biological repeats were performed, this is noted in the figure legends
Randomization	Randomization was not required for the type of in vitro data we have reported in this study
Blinding	No blinding was used in this study

Reporting for specific materials, systems and methods

We require information from authors about some types of materials, experimental systems and methods used in many studies. Here, indicate whether each material, system or method listed is relevant to your study. If you are not sure if a list item applies to your research, read the appropriate section before selecting a response.

Materials & experimental systems

n/a	Involved in the study	
[ ]	[x]	Antibodies
[ ]	[x]	Eukaryotic cell lines
[x]	[ ]	Palaeontology and archaeology
[x]	[ ]	Animals and other organisms
[x]	[ ]	Human research participants
[x]	[ ]	Clinical data
[x]	[ ]	Dual use research of concern

Methods

n/a	Involved in the study	
[x]	[ ]	ChIP-seq
[ ]	[x]	Flow cytometry
[x]	[ ]	MRI-based neuroimaging

Antibodies

Antibodies used

BRCA1 OP92 Calbiochem 1/200
BRCA2 OP95 Calbiochem 1/500
ACTIN ab8226 Abcam 1/1000
FLAG F3165 Sigma 1/1000
PCNA ab18197-100 Abcam 1/1000
Histone H3 ab1791 Abcam 1/1000
γH2AX (S139) (JBW301) 05-636 Millipore 1/1000
PAR Polymer 4335-MC-100 Trevigen 1/1000
PARP1 9542 Cell signalling 1/1000

SIRT1 8469S Cell signalling 1/1000
SIRT3 2627S Cell signalling 1/1000
SIRT6 12486S Cell signalling 1/1000
pRPA32 (S4/S8) A300-245A Bethyl 1/1000
HPF1 NBP1-93973 NovusBiologicals 1/1000
Acetylated-Lysine 9441S Cell signalling 1/1000
BrDU 347580 BD Biosciences 1/100

Validation	Antibodies were all validated by commercial source. No homemade or previously unpublished antibodies were used in this study. All antibodies for proteins or protein-modifications included in this study were validated using western blotting and/or immunofluorescence staining.

Eukaryotic cell lines

Policy information about cell lines

Cell line source(s)	SUM149 cells (Asterand Bioscience), DLD1 BRCA2 +/+ and –/– (Horizon Discovery), HAP1 WT, SIRT1–/–, SIRT3 –/–, SIRT6–/– (Horizon Discovery), CAL51 (DSMZ), HEK293T, MDA-MB-436 (ATCC) and U2OS WT and U2OS HPF1–/– (gift from Ivan Ahel)
Authentication	All human cell line identities used in this study were confirmed by Short tandem repeat (STR) typing.
Mycoplasma contamination	All human cell lines used in this study were routinely (monthly) verified free of mycoplasma infection using Lonza MycoAlert.
Commonly misidentified lines (See ICLAC register)	No commonly misidentified cell lines were used in this study.

Flow Cytometry

Plots

Confirm that:

- [x] The axis labels state the marker and fluorochrome used (e.g. CD4-FITC).
- [x] The axis scales are clearly visible. Include numbers along axes only for bottom left plot of group (a 'group' is an analysis of identical markers).
- [x] All plots are contour plots with outliers or pseudocolor plots.
- [x] A numerical value for number of cells or percentage (with statistics) is provided.

Methodology

Sample preparation	Cells were fixed in ice-cold 70% (v/v) ethanol and stored at −20°C until use (see full description in the materials and methods section of this manuscript).
Instrument	LSRII was used for data aquisition
Software	Flow cytometry data analysis was performed using FlowJo v10
Cell population abundance	Gating for single cells was performed for each sample analysed. Cell abundance corresponding to each cell cycle phase was determined by equal application of gates (see below) and division with the total number of cells analyzed for each sample respectively.
Gating strategy	Using Propidium iodide (PI) and Alexa 488 labelled BrdU we were able to gate the cell cycle phases in agreement with previously published reports. We denoted cells with BrdU high as S-phase, BrdU low/PI low as G1-phase, and BrdU low/PI high as G2-phase cells respectively.

- [x] Tick this box to confirm that a figure exemplifying the gating strategy is provided in the Supplementary Information.
